# A Systematic Review of Zoonotic Enteric Parasites in Synanthropic Mammalian Species in Florida

**DOI:** 10.3390/pathogens13121065

**Published:** 2024-12-03

**Authors:** Jeffrey M. Perera, Claire Gurtler, Amber N. Barnes

**Affiliations:** 1 Department of Biology, University of North Florida, Jacksonville, FL 32224, USA; 2 Department of Biological Sciences, Vanderbilt University, Nashville, TN 37235, USA; 3 Department of Public Health, University of North Florida, Jacksonville, FL 32224, USA

**Keywords:** Parasitology, one health, zoonoses, wildlife, infectious disease, gastrointestinal disease, epidemiology, mammals

## Abstract

Florida is home to a vast number of wildlife species that come into close contact with residents and domestic animals. As mammals are competent hosts for many zoonotic diseases, it is important to understand what exposure risks are present for both people and animals. Using Preferred Reporting Items for Systematic reviews and Meta-Analyses (PRISMA) guidelines, this review analyzed published literature spanning 1963 through 2023 documenting zoonotic enteric parasites in synanthropic wild mammals of Florida, excluding mice and rats. Between an initial search conducted in 2022 and updated search in 2024, 10,563 titles were reviewed. Using predetermined inclusion and exclusion criteria, 26 titles were included in the final analysis examining a range of acanthocephala, cestode, nematode, protozoa, and trematode parasite species. Of the results, most studies found at least one parasite in Florida raccoons (n = 14) with additional studies in opossums (n = 8), armadillos (n = 4), bobcats (n = 4), coyotes (n = 3), squirrels (n = 3), gray foxes (n = 1), red foxes (n = 1), an undeclared fox type (n = 1), and bats (n = 1). No studies were found documenting zoonotic enteric parasites in rabbits or moles. The transmission pathways for each parasite and the zoonotic exposure risks varied significantly. Coordinated One Health prevention and control efforts must be targeted for effectiveness.

## 1. Introduction

It has been estimated that the state of Florida is home to more than 500 species of marine mammals and fish, 200 species of freshwater fish, and more than 700 species of land animals [1]. The unique natural habitats for this wide variety of fauna often overlap with residential areas, leading to contact between wild animals, domestic animals, and people. Some wildlife have learned to take advantage of this contact for access to food sources and shelter. Synanthropic animals are a diverse range of wild species that have adapted to living in close proximity to humans and their built environments, spanning both rural and urban areas [2]. However, as more than 60% of all human infectious diseases are zoonotic in origin, these animals may play a critical role in the spread of zoonotic parasites to humans and other animals [3,4].

The transmission of zoonotic enteric parasites (ZEPs) to humans can occur through multiple exposure pathways such as the ingestion of contaminated food, water, or soil, as well as through the consumption of raw or undercooked infected meat, aquatic organisms and seafood, or other animal-derived products [4]. Additional exposure risks are associated with contact with contaminated soil or recreational water, unclean hands, and insect vectors that breed and feed on excreta and waste, such as flies and cockroaches [4,5]. People may also be at risk of exposure when pets hunt or interact with infected mammals, amphibians, reptiles, or birds [6]. Pets who come into contact with infected wildlife can spread zoonotic diseases to their household, or to other animals at their home site via saliva, aerosols, contaminated urine or feces, and direct contact [7,8].

In Florida, synanthropic animals such as rodents and other small mammals, including mid-sized carnivores, can be found living in or moving across areas where unique ecosystems meet, such as forests, wetlands, marshes, beaches, and residential or other developed areas [2,9]. For this reason, understanding the complex involvement of ZEPs at the human–animal interface is critical for protecting both public health and animal health. This research can inform the development of effective measures to prevent the transmission of enteric parasites to humans from wildlife, and vice versa [10]. While much research has been conducted on the zoonotic disease potential of contact with rodents such as mice and rats in both urban and residential areas, there is less information regarding other synanthropic mammals that are common in Florida, including squirrels [11,12,13]. According to the Florida Fish and Wildlife Conservation Commission (FWC), residents across Florida have frequent residential contact with the following additional mammals (excluding mice and rats): armadillos, opossums, raccoons, eastern moles, bats (especially the Brazilian free-tailed bat), eastern cottontails, gray squirrels, red foxes, gray foxes, coyotes, and bobcats (T. Doonan, personal communication, 11 August 2022). These synanthropic mammals have the potential to spread ZEPs to humans and domestic animals in their shared environment. Therefore, the purpose of this study was to investigate the prevalence of zoonotic enteric parasites in synanthropic mammals of Florida (excluding mice and rats) while identifying common exposure risks and One Health-related prevention and control measures to avoid zoonotic and reverse zoonotic infection.

## 2. Materials and Methods

### 2.1. Search Strategy

Between 6 October and 26 October 2022, an initial search of the following electronic databases was conducted: PubMed; Web of Science Core Collection; Google Scholar; Environment Complete; ScienceDirect; GALE databases of Agriculture Collection, Nursing and Allied Health Outcomes, and Environmental Studies and Policy; and the ProQuest databases of the ABI/INFORM Collection, Agricola, Earth, Atmospheric, and Aquatic Sciences Collection, Agriculture, and Environmental Science Collection, Health and Medicine, MEDLINE (Proquest), and TOXLINE.

Using the Preferred Reporting Items for Systematic reviews and Meta-Analyses (PRISMA) 2020 search and screening guidance, a search string was comprised of terms related to synanthropic mammals in Florida including armadillos, opossums, raccoons, eastern moles, bats (especially the Brazilian free-tailed bat), eastern cottontails, gray squirrels, red foxes, gray foxes, coyotes, and bobcats in conjunction with ZEPs [14]. Rats and mice were excluded from this study. If the option was available, the search results were filtered to include only journal articles written in the English language and keywords mentioned in the title and abstract. There was no time restriction for this search. Accessible results were copied into the citation manager RefWorks (ProQuest LLC, Ann Arbor, MI, USA) by database, and a master folder was created for all titles found in the initial search.

An updated search was conducted between 16 and 23 January 2024, using the same parameters as above. Titles published between the original search dates in October 2022 and 31 January 2024 were recorded for analysis. An informal protocol with the full listing of all search strings used, dates searched, and their corresponding database results is provided in the Supplementary Materials [S1]. The review was registered with PROSPERO prior to the initial search [PROSPERO registration CRD42022360795].

### 2.2. Criteria for Inclusion and Exclusion

Inclusion criteria consisted of titles that were (a) peer-reviewed journal articles, including articles with English abstracts; (b) from any publication year; (c) observational studies (i.e., cohort studies, case–control studies, and cross-sectional studies); (d) conducted in armadillos, opossums, raccoons, eastern moles, bats, eastern cottontails, gray squirrels, red foxes, gray foxes, coyotes, and bobcats (e) naturally infected in Florida with a documented presence of (f) zoonotic parasites that have a primarily enteric transmission route and are of human health importance.

Exclusion criteria comprised (a) any publication that was not a peer-reviewed journal article; (b) publications written in languages other than English; (c) any publication that did not include primary research or was not an observational study (e.g., experimental studies, systematic reviews, meta-analyses, other review articles, letters to the editor, conference papers, thesis and dissertations, technical reports, working papers, white papers, conference proceedings, preprinted formal articles, and patents); (d) research on an animal species outside of the curated list; (e) research in which the animal(s) sampled did not originate in Florida, USA; (f) research that only presented null results; or (g) research in which the parasites are not generally considered zoonotic or likely to be zoonotic. If a title mentioned zoonotic enteric parasites that were not included in the initial list of search terms, it was included in the study if the team agreed upon review that it met the criteria outlined above.

### 2.3. Screening Process and Study Selection

Following PRISMA guidelines, articles were screened first for eligibility based on the content of the journal article titles and abstracts [14]. Titles and abstracts were assessed for eligibility by one reviewer, while a quality control check on at least 25% of the title and abstracts to ensure accuracy in the screening was performed by a second reviewer. Titles that were not clearly outside of the search parameters were conservatively included in the initial screening and were fully assessed during the full-text review. Full-text copies were obtained for all available titles that met the inclusion criteria in the title- and abstract-screening phase. Each full-text title was reviewed by two reviewers based on the eligibility criteria above and subsequently marked for inclusion or exclusion, ensuring that they made note of their justification. If a unanimous decision had not been formed for each title, the third co-author would have been asked to serve as the tiebreaker. If multiple titles pertained to the same study or dataset, only the one that provided the most complete information was included in the analysis.

### 2.4. Data Extraction

During the full-text review, initial notes were taken by both reviewers on studies that met the inclusion criteria for categories including animal species, zoonotic enteric parasite(s), Florida county(s), prevalence rate(s) or percent of samples that tested positive, risk factors for transmission, and recommendations for the prevention of disease within human and animal populations. This information was gathered from the document text, tables, and/or graphs as presented by the original author(s). If risk factors for human–animal transmission or recommendations for the prevention of disease were not discussed by the original author(s) in the included title, additional resources were utilized and cited accordingly.

### 2.5. Heatmap Figure Generation

A heatmap illustration was generated to visualize the percent positives for parasites found in racoons, according to the included studies, using Python programming language software version 3 (Python Software Foundation, Wilmington, DE, USA https://www.python.org accessed on 1 September 2024). The libraries Matplotlib [15] and seaborn [16] were used to generate the heatmap. Data from each study were loaded and processed using the Pandas library [17]. Parasite prevalence values were annotated directly onto the heatmap to help visualize the prevalence rates across studies. A custom color map (“gist_heat_r”) was applied to accentuate variations in parasite prevalence rates, emphasizing values ranging from 0 to 100 percent. The color bar was adjusted to visually distinguish parasite hotspots and scaled with a center at 60%.

## 3. Results

**Figure 1:**
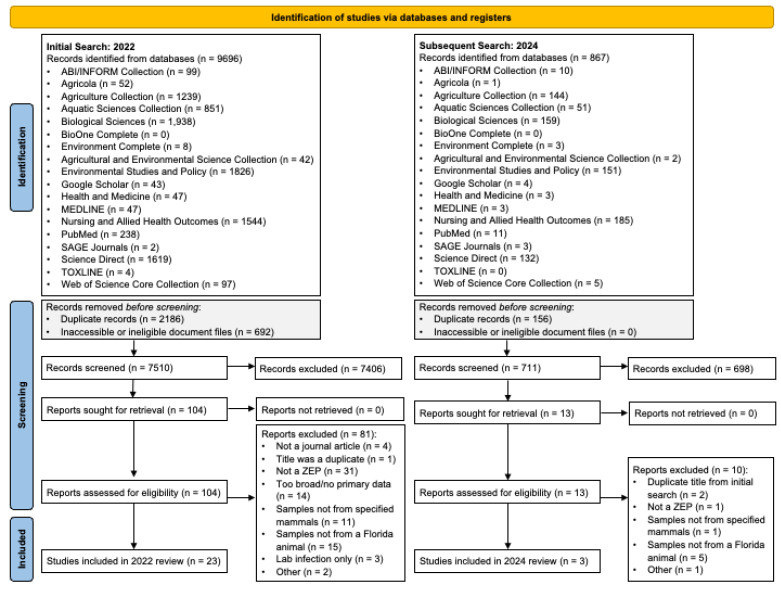
PRISMA screening flowchart of study selection and inclusion.

The initial search led to 9696 title results across all databases (Figure 1). Of these titles, 2186 were either duplicates or inaccessible records. The remaining 7510 records were then originally screened by one reviewer for consistency; of these, only 104 records met the described criteria for full-text review. Full-text screening was performed by two reviewers, who were in agreement to remove a further 81 records for not meeting the inclusion criteria, as they were not a journal article (n = 4), the record was a missed duplicate title (n = 1), the parasite was not zoonotic and/or enteric (n = 31), the record was a review without primary data (n = 14), the study animals were not the species outlined in our search (n = 11), the samples collected were not from a Florida animal (n = 15), the presence of the parasite was not due to natural infection (n = 3), or for other reasons (e.g., there were no positive findings; n = 2). This resulted in an initial 23 articles for inclusion.

In the subsequent search, 867 more titles were found across all databases (Figure 1). After removing 156 duplicates, 711 titles remained for title- and abstract-screening by the same single reviewer who completed this step in the initial search. Full-text screening was then conducted on the 13 titles by two reviewers, as previously described. At this stage, 10 titles were excluded for the following reasons: duplicate title included in the initial search (n = 2), the parasite was not zoonotic and/or enteric (n = 1), the study animals were not the species outlined in our search (n = 1), the samples collected were not from a Florida animal (n = 5), or for another reason (e.g., there were no positive findings; n = 1). An additional three articles were included for analysis after this follow-up search. In total, 26 titles were included in the study results.

**Figure 2:**
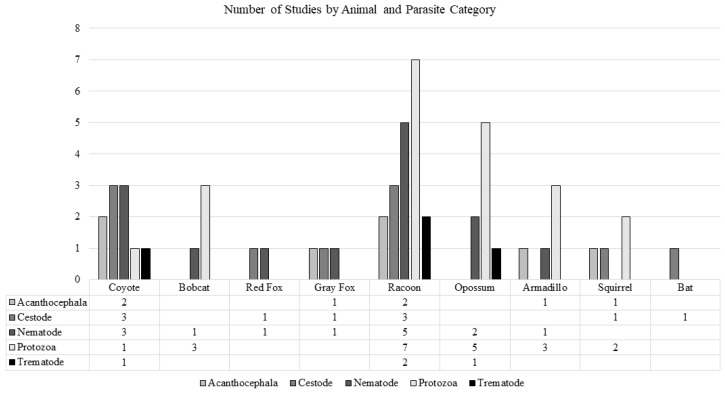
The number of included studies by animal species and parasite category. Several studies investigated multiple animals and parasites. An additional study found ZEPs in an unspecified Florida fox species [18].

The findings included unique studies in raccoons (n = 14), opossums (n = 8), armadillos (n = 4), bobcats (n = 4), coyotes (n = 3), squirrels (n = 3), gray foxes (n = 1), red foxes (n = 1), an undeclared fox type (n = 1), and bats (n = 1; Figure 2). Some studies were conducted in multiple animal types and/or examined multiple parasite species. No studies on zoonotic enteric parasites in Florida’s rabbits or eastern moles were found in this search. Many species of ZEPs were identified by the study authors in the categories of acanthocephala, cestode, nematode, protozoa, and trematode. Several studies reported additional parasites with no known zoonotic potential. These parasites were not included in this review.

Among the positive samples presented within the included articles, the Acanthocephala species included an unidentified *Acanthocephala* sp., *Macracanthorhynchus ingens*, *Moniliformis clarki*, and *Moniliformis moniliformis* (Table 1). The cestode species included *Diphyllobothrium latum*, *Dipylidium caninum*, *Hymenolepis* spp., *Mesocestoides* spp., *Mesocestoides variabilis*, *Raillietina bakeri*, *Spirometra mansonoides*, and an unidentified *Taenia* sp. The nematode species included *Ancylostoma braziliense*, *Ancylostoma caninum*, *Angiostrongylus cantonensis*, *Angiostrongylus costaricensis*, an unidentified *Ascaris* sp., *Baylisascaris procyonis*, *Capillaria aerophila*, *Capillaria plica*, *Capillaria putorii*, an unidentified *Capillaria* sp., *Gongylonema pulchrum*, an unidentified Spirurid nematode sp., an unidentified *Strongyloides* sp., *Strongyloides stercoralis*, *Toxocara canis*, *Trichinella spiralis*, *Trichuris vulpis*, and *Uncinaria stenocephala.* The protozoa species included *Balantidium coli*, *Blastocystis* sp., *Cryptosporidium* spp., *Cytoisospora* spp., *Entamoeba hystolytica*, *Trypnasoma cruzi*, an unidentified *Sarcocystis* sp., and *Toxoplasma gondii.* The trematode species included an unidentified *Alaria* sp., *Fibricola cratera*, *Heterobilharzia americana*, and an unidentified *Paragonimus* sp.

Within the coyotes of Florida, 20 unique parasites species were found from the categories of acanthocephala [19,20], cestode [19,20,25], nematode [19,20,25], protozoa [25], and trematode [25]. Of the Florida bobcats sampled within the included studies, researchers found four parasitic nematodes [27] and protozoa [38,40,41] species. In red foxes, six different cestode [19] and nematode [19] species were documented, while in gray foxes, 11 parasites from the acanthocephala [19], cestode [19], and nematode [19] categories were highlighted. An additional study population of “foxes” in Florida were also positive for nematodes [18]. Among smaller mammals, racoons exhibited a variety of 15 parasite species studies in the included publications, such as those from the acanthocephala [21,22], cestode [21,22,26], nematode [18,22,29,30,31], protozoa [33,34,35,36,39,40,42], and trematode [21,22] categories. Opossums were found to have five parasite species from the nematode [18,29], protozoa [34,35,36,37,40], and trematode [43] categories. Armadillos contained four distinct parasites from the acanthocephala [23], nematode [28], and protozoa [28,36,40] categories. Squirrels were also reported to have four unique parasites from the acanthocephala [24], cestode [24], and protozoa [36,40] categories. Florida bats were found to contain one zoonotic cestode parasite [32]. No studies were found that documented ZEPs in rabbits or moles.

**Figure 3:**
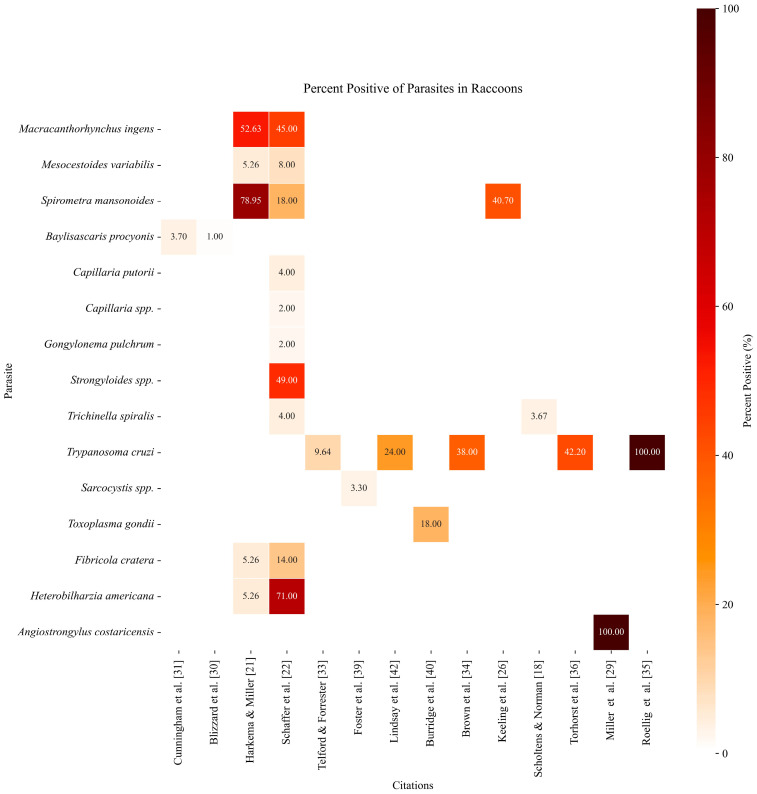
Heatmap of percent positive for racoon parasites. Note: Prevalence values of 100% reflect data from small sample sizes and may not be representative of prevalence among the broader raccoon population [18,21,22,26,29,30,31,33,34,35,36,39,40,42].

The heatmap graphic represents the prevalence of various parasites in raccoon populations across studies that were included after full-text screening (Figure 3). Each cell displays the percentage of raccoons that tested positive for a given parasite in specific studies, with the intensity of the color corresponding to the percentage of positivity described in the study. Darker colors represent a higher percentage of infection, while lighter colors correspond to a lower prevalence.

The analysis of the data included in the heatmap figure highlights *Macracanthorhynchus ingens*, *Spirometra mansonoides*, *Strongyloides* spp., *Trypanosoma cruzi*, *Heterobilharzia americana,* and *Angiostrongylus costaricensis* as the parasites with the highest mean prevalences across the studies of Florida raccoons. Overall, the mean prevalence of parasites in raccoons across all studies ranged between 1% and 100%, depending upon the parasite species and sample size, with an average of 27.7%. This underscores the variability in parasite–host relationships within raccoon populations and emphasizes the importance of understanding zoonotic risks associated with specific parasite species.

## 4. Discussion

The results of this study demonstrate a wide variety of zoonotic enteric parasites circulating within Florida’s coyotes, bobcats, red and gray foxes, racoons, opossums, armadillos, squirrels, and bats. For most of the ZEPs outlined in the publications, the greatest exposure risks for people were through accidental fecal–oral transmission or the ingestion of contaminated food, drink, soil, or vectors. However, the roles of human, animal, and environmental reservoirs as well as intermediate, definitive, paratenic, and “dead-end” hosts varies significantly by parasite. Understanding more about the parasite life cycles and zoonotic, and reverse zoonotic, exposure risks can help public health and veterinary health practitioners to design and implement successful prevention strategies in a One Health framework.

### 4.1. ZEPs Transmitted Through Fecal–Oral Pathways

*Ascaris* spp. were found in coyote samples [25], although humans and pigs are the most common hosts for these nematodes [44]. Roundworm eggs are deposited into the environment through poorly managed and untreated fecal waste [44]. These eggs are then ingested due to contamination of food, water, hands, soil, or objects [44]. *Baylisascaris procyonis* is another ZEP found in the included article results within Florida racoons [30,31]. *B. procyonis* is known as the racoon roundworm but, despite racoons serving as the definitive host for this parasite, dogs and other canids can serve as effective alternative hosts [44]. These animals become infected after eating smaller paratenic hosts, such as rodents or birds [44]. Humans are accidental hosts who are exposed via the consumption of infected parasite eggs in food, water, or soil or through contaminated hands and other objects [44]. Likewise, the roundworm *Trichuris vulpis* appears to infrequently infect people [45,46]. Instead, this nematode is often associated with dogs, wolves, and coyotes. The definitive canid host sheds eggs in their feces that are then ingested by a new host, with human exposure likely due to contact with infected dogs [45,46].

The protozoan *Balantidium coli*, also taxonomically known as *Neobalantidium coli* or *Balantioides coli*, is also spread through fecal contamination of the environment, leading to the consumption of contaminated food or water by a new host [44]. Typical reservoirs include pigs, rodents, people, and other primates; however, *Balantidium coli* has been reported in Florida coyotes [25,44]. *Cystoisospora* spp., *Cryptosporidium* spp., and *Entamoeba histolytica* were also recorded in coyotes in the state [25]. Cystoisosporiasis in people is associated with accidental fecal–oral ingestion, usually associated with immunocompromised individuals or people in vulnerable, crowded settings [44]. Cryptosporidiosis, however, is a common zoonotic protozoan that infects people and various animals, including mammals, birds, reptiles, and amphibians [44]. Infected oocysts are shed in stool that can then contaminate food, water, soil, hands, objects, etc. Exposure occurs after accidental fecal–oral ingestion through one of these routes [44]. Environmental contamination with *Entamoeba histolytica* occurs when infectious cysts and trophozoites are excreted in feces [44]. *E. histolytica* is then primarily transmitted via contaminated food, water, soil, hands, objects, etc. Exposure to feces during sex can also be a risk factor for many enteric diseases, including *E. histolytica* [44].

### 4.2. ZEPs Transmitted Through Ingestion of a Vector

For many of the identified ZEPs in this review, arthropods and other insects play a significant role in transmission to both animals and people. For example, Conti et al. identified *Moniliformis clarki* in Florida squirrels [19]. While not a common zoonotic species, a pediatric case-report of an infant in Florida with failure-to-thrive was eventually determined to be an infection with *M. clarki*, likely due to accidental cockroach ingestion [47]. The natural definitive host for acanthocephalan parasite species varies: raccoons for *Macracanthorhynchus ingens* and rats for *Moniliformis moniliformis* [44]. But within this review, *M. ingens* was found not only in Florida racoons [21,22], but also in armadillos [23] and coyotes [20]. Additionally, *M. moniliformis* was found in Florida gray foxes [19]. These hosts can shed infected parasite eggs into the environment that are subsequently ingested by an arthropod intermediate host, such as a cockroach or beetle [44]. Human transmission is often due to contact with animals or the consumption of insects harboring cystacanths, the infectious stage of the parasite [44].

Arthropods also serve in the transmission cycle for the “gullet worm” or “stitch worm”—*Gongylonema pulchrum* [44]. Found in Florida racoons [22], the typical definitive hosts are livestock and wild ungulates [44]. Infected parasite eggs are passed in feces into the shared environment. Intermediate insect hosts, such as cockroaches and dung-feeding beetles, facilitate the life cycle of the parasite. Humans are exposed, much like the definitive hosts, by consumption of an infected arthropod [44]. A similar transmission cycle is found with *Raillietini* spp. [44]. Within the cestode ZEPs outlined in the review, *R. bakeri* was found in a squirrel [24]. *Raillietini* spp. infection needs definitive hosts of rodents or birds, depending on the species, but also intermediate hosts of either beetles or ants [44]. Transmission occurs after ingesting an infected intermediate host. Parasitic eggs are then shed in the feces of the definitive host, including humans, until being ingested by another susceptible intermediate host [44].

*Dipylidium caninum* and *Hymenolepis* spp. tapeworms were reported in Florida coyotes [25]. *Dipylidium* infection in both animals and people is due to the ingestion of fleas that harbor tapeworm larvae (cysticercoids), often through grooming on the part of animals [44]. Segments of the tapeworm are passed in feces and, once in the environment, they serve as a food source for flea larvae [44]. Hymenolepis infection, on the other hand, can be transmitted to people through accidental fecal–oral ingestion of tapeworm eggs via contaminated food, water, hands, soil, or consumption of the intermediate insect hosts, depending on the cestode species [44].

An additional insect vector that aids in the spread of a zoonotic parasite is the triatomine, or “kissing bug.” Bites from infected triatomine bugs are the primary exposure pathway for *Trypanosoma cruzi* [44]. The vector first bites an infected host and then, via a second blood meal, they transmit the parasite to a new host. Common animal reservoir hosts for *T. cruzi* include rodents, dogs, armadillos, opossums, and racoons [44]. However, transmission can also occur due to organ transplants, blood transfusions, and consumption of the vector itself or triatomine bug feces in contaminated food or water [44]. In this review, *T. cruzi* was documented in Florida racoons [33,34,35,36], opossums [34,35,36,37], armadillos [36], and squirrels [36].

### 4.3. ZEPs Transmitted Through Ingestion of Meat, Fish, and Other Tissues

*Mesocestoides* spp., including *M. variabilis*, were identified in gray foxes [19] and racoons of Florida [21,22]. Much like other tapeworms, infectious material is shed in feces then first ingested by arthropod and/or other insect intermediate hosts. Insects infected with oncospheres are then consumed by second intermediate hosts, including amphibians, reptiles, rodents and other small mammals, and birds, where tetrathyridia develops [44]. In this stage, the tapeworm begins to infect organs. Definitive hosts include dogs, foxes, raccoons, opossums, cats, and more that feed on the intermediate prey and are exposed through eating infected tissue [44]. Humans are also infected through consumption of the tissue, meat, or viscera of animals with tetrathyridia [44]. People are the definitive host for several *Taenia* spp. and pass the tapeworm eggs in their stool [44]. With poorly managed sanitation services, the eggs can contaminate grazing areas for pigs and cattle. Once these animals are infected, the tapeworm larvae (cysticerci) move to their muscle tissue. Human transmission occurs after ingestion of infected meat tissue that is served raw or undercooked [44]. The same is true of sarcocystosis parasitic infection. Humans are then exposed by eating the undercooked tissue, meat, or viscera of infected animals [44]. In this review, *Sarcocystis* spp. were documented in both bobcats [38] and racoons [39], although unlikely zoonotic species.

Trichinellosis, another human parasitic infection, is also caused by the consumption of undercooked or raw tissue, meat, or viscera [44]. *Trichinella spiralis* was recorded in Florida foxes [18], racoons [18,22], and opossums [18]. Because the larva encysts into the muscle of the host, ingestion is the route of exposure for both definitive and intermediate hosts [44]. Depending on *Trichinella* spp., animal transmission occurs in either a domestic cycle, often with small rodents and pigs, or a sylvatic cycle, frequently associated with bears, moose, wild boars, and small rodents [44]. The definitive/intermediate hosts may be exposed through eating the tissue of other infected animals [44].

Toxocariasis also occurs in people after the ingestion of parasite eggs, larvae, or paratenic host tissue [44]. In this study, *Toxocara canis* was found in coyotes [25] and red foxes [19]. *T. canis* is naturally associated with all canids [44]. Parasite eggs are shed in feces and once in the environment, they are consumed by paratenic hosts such as rabbits, ducks, birds, and livestock [44]. Transmission to definitive hosts can occur after eating the undercooked or raw tissue, meat, or viscera of infected paratenic hosts or the infected eggs themselves [44]. Furthermore, toxoplasmosis infection can happen after consuming undercooked or raw tissue, meat, or viscera [44]. While the definitive animal hosts for *Toxoplasma gondii* are cats and other felids, intermediate hosts such as birds and rodents are infected by ingesting food, water, or soil that is contaminated by cat feces [44]. This is another exposure route for humans, along with blood transfusion, organ transplants, vertical transmission, or direct environmental exposure to cat feces [44]. Cats are exposed to *T. gondii* after eating infected intermediate hosts.

Much like the risk of eating meat products, intestinal capillariasis, caused by infection with *Capillaria* spp. roundworms, can be transmitted through the consumption of raw or undercooked fish containing infective larvae [44]. Eggs are shed in feces that, when in water, continue the parasite’s life cycle. Upon being eaten by a fish, the larvae migrate to its tissue. The parasite is then transmitted to a human host through the ingestion of infected fish [44]. In this current review, *Capillaria* spp. were found in racoons [22] and bats [32]. More specifically, *C. aerophila* were documented in coyotes, red foxes, and gray foxes [19], *C. plica* in coyotes [19], and *C. putorii* in racoons [22]. These three *Capillaria* spp. have also been documented in humans [48,49].

Human diphyllobothriid infection can occur from consumption of undercooked or raw freshwater fish, such as salmon or trout [44,50]. The definitive hosts for the parasite *Diphyllobothrium latum* includes mammals (e.g., dogs, foxes, coyotes, etc.) and fish-eating birds [44,50]. *D. latum* was reported in a study among Florida coyotes [25]. Infected feces deposited near water bodies begin the *D. latum* life cycle with eggs growing into swimming larvae (coracidium) ingested by the first intermediate host, crustaceans. Here, they develop into procercoids that when ingested by a second intermediate host(s)—fish—grow into the plerocercoid stage. When once more ingested by either larger predatory fish or another definitive host (e.g., humans), they mature into adult tapeworms. Similarly, *Spirometra* spp. use multiple types of aquatic hosts, although typical definitive hosts of *Spirometra* spp. are dogs and cats. When near water, hosts shed infected eggs in their feces [44]. These eggs are ingested by crustaceans as first intermediate hosts and then by larger, aquatic second intermediate hosts (e.g., amphibians, reptiles, and fish) [44]. Transmission to animals can occur when they eat the second intermediate host. *S. mansonoides* was reported in this review in both gray foxes [19] and racoons of Florida [21,22,26]. Human exposure can come from either the consumption of undercooked or raw infected crustaceans (first intermediate host), fish, reptiles, or amphibians (second intermediate hosts), or contaminated water [44]. Likewise, intentionally eating undercooked or pickled crustaceans, such as crab or crayfish, infected with *Pargonimus* spp. may expose people or other animal hosts, including pigs, dogs, felines, and more [44]. Trematode eggs are shed from an infected host and once in water, they begin their life cycle that uses two intermediate hosts—first snails and then crustaceans [44]. It is the ingestion of infected intermediate crustacean hosts that perpetuates the parasite transmission in people and animals [44]. In Florida, *Pargonimus* spp. were noted in coyotes [25].

Gnathostomiasis, caused by infection with nemotades such as *Gnathostoma* spp., also starts with parasite eggs shed in feces that then hatch in water. They are first ingested by copepods and then these copepods are ingested by a second intermediate host, for example, fish or amphibians [44]. Definitive parasite hosts, such as canids, felines, and omnivorous mammals, then consume the infected intermediate hosts. On the other hand, the infected intermediate hosts could also be consumed by paratenic hosts like birds and snakes [44]. Human infection occurs via consumption of undercooked or raw tissue or meat from the infected intermediate host (e.g., fish and amphibians) or paratenic hosts (e.g., birds and snakes) [44]. In this review, spirurid nematode larvae of unknown species were reported in red and gray foxes in Florida [19]. The *Alaria* spp. of trematodes may also cause exposure to people through the ingestion of infected paratenic hosts. Consumption of undercooked amphibians, particularly frog legs, was identified as a likely source of intraocular infection with *A. mesocercaria* [51]. *Alaria* spp. were reported in coyotes within this review [25]. Another uncommon yet potential zoonotic trematode of public health concern is *Fibricola cratera*, documented in both Florida racoons [21,22] and opossums [43]. Experimental human infection with *F. cratera* demonstrated similar symptoms to another zoonotic *Fibricola* spp., *F. seoulensis*, which has been transmitted to people through the ingestion of paratenic reptiles and amphibians [52,53].

Ingestion of other infected tissues, such as gastropods, may also serve as an exposure route to *Angiostrongylus* spp. roundworms. Both *A. cantonensis* and *A. costaricensis* species depend on rats as their definitive host, which shed eggs in their feces [44]. Both species also use a gastropod as an intermediate host: *A. cantonensis* prefers snails, while *A. costaricensis* prefers slugs [44]. Florida synanthropic mammals have demonstrated *Angiostrongylus* spp. infection, including *A. cantonensis* in armadillos [28] and *A. costaricensis* in racoons and opossums [29]. Humans and susceptible rodent hosts are infected by accidental consumption of the gastropod or infectious larvae in contaminated fruits and vegetables [44].

### 4.4. ZEPs Transmitted Through Direct Contact with Contaminated Environment

Several hookworm parasites found in Florida wildlife are shed into the environment through the infected feces of definitive hosts but otherwise use direct contact for transmission. Depending on the hookworm species, the definitive hosts may be canids (e.g., foxes, coyotes, and dogs), felids (e.g., cats), or both. The larvae do not generally cause intestinal illness in new susceptible hosts [44]. Instead, the larvae pierce the skin of potential hosts [44]. In the studies included in this review, these hookworm species included *Ancylostoma braziliense* in bobcats [27] and gray foxes [19], *Ancylostoma caninum* reported in coyotes [19,20,25], bobcats [27], and red and gray foxes [19], and *Uncinaria stenocephala* in coyotes [25].

Strongyloidiasis in humans is also spread through larvae piercing the skin of a definitive host, such as humans, dogs, or other primates—depending on the *Strongyloides* spp. [44]. When infected stool from a host is deposited in the environment, direct contact with the contaminated soil can expose a new host to these “threadworms” [44]. In this review, *Strongyloides* spp. were found in Florida racoons [22] and more specifically, *S. stercoralis* was reported in gray foxes [19]. In addition, the schistosome parasite *Heterobilharzia americana* is spread through infected eggs deposited in feces from the definitive host, usually a bird but also dogs and racoons [44]. Once in water, the eggs find intermediate molluscan hosts to help them in developing into cercariae that find new susceptible hosts. Human infection occurs when the cercariae penetrate the skin, causing “swimmer’s itch” [44].

Unlike the ZEPs listed above, the life cycle of *Blastocystis* spp. is less well understood. These parasites colonize the gastrointestinal tracts of people and many animals, but the pathogenicity is unknown [44]. In this study, *Blastocystis* spp. were reported in coyotes [25].

Understanding more about the epidemiology of zoonotic parasites can help researchers and providers outline transmission pathways to design opportunities for prevention and control. This is critical because the synanthropic nature of the animals highlighted in this study presents an exposure risk to humans and other animals in residential and urban settings. For example, coyotes have experienced significant expansion across all areas of the United States with more recent movement into Florida [54,55]. While coyotes still generally prefer natural habitats away from humans, anthropogenic food sources (including pets) have resulted in growing urban populations of coyotes [56,57,58]. And while the risk of human–animal conflict remains low, the bigger risk may be in zoonotic disease transmission [56,59,60]. Coyotes and other types of carnivores use excreta, such as scat, for intraspecies communication. This leads to feces and urine deposition in purposeful locations, including areas commonly utilized by humans and pets [60]. Additionally, coyotes are omnivorous and eat a mixture of food items such as insects, fruits/berries, small rodents or birds, and even larger ungulates like deer or livestock [61]. Coyotes living near human settlements may also eat garbage, leftover pet food, and even small pets [61]. Securing garbage cans, livestock, poultry, and companion animals near home sites, coupled with removing pet food and dropped fruit, can help to deter coyote interactions and lessen zoonotic transmission risks near home sites [61].

Bobcats are also commonly found throughout Florida. And while they, too, maintain a preference for natural habitats, they are adaptable to residential areas [62,63]. The range of bobcats can span several miles, including up to 6 square miles in rural areas and up to 2 miles in urban zones [61]. This range can successfully overlap areas where other native mammals exist, such as coyotes, leading to contact between the species [54]. And although coyotes in Florida consume a variety of food sources, bobcats are carnivores. Within the state, they consume primarily small rodents, squirrels, and rabbits—again introducing many opportunities for multi-species contact [54,61]. However, opportunistic residential bobcats have been known to hunt chickens or small pets [61]. Like coyotes, recognizing how to discourage bobcat activity near a home can assist in ZEP prevention.

Red and gray foxes may be found near Florida households, too. Red foxes have only more recently expanded their range across Florida, yet gray foxes have long been considered a native species [61]. Both fox types are primarily nocturnal and omnivores, with diets spanning small rodents and rabbits, birds, reptiles, amphibians, fish, insects, fruit/berries, and even carrion [61,64]. If given the chance, foxes may pursue chickens or other poultry at home sites. Yet due to their size, foxes often serve as prey for coyotes, bobcats, dogs, and other animals [61]. Preventing fox–human or fox–pet contact can reduce the zoonoses exposure risks to all.

Additional mid-sized mammals that could expose people and domestic or companion animals to parasites include racoons and opossums. Racoons are intelligent and prefer to live near food and water sources, often turning to urban areas for both [61]. With a preference for fruit and food scraps, trash, eggs, smaller animals (e.g., rodents), insects, and crustaceans, residential habitats can be enticing [61,65]. Pet food and even bird feeders can attract raccoons [65]. Similarly, opossums are common in urban areas across Florida due to the plethora of food sources including gardens, garbage, pet food, fruit trees, and more [61]. However, opossums also scavenge carrion and roadkill, introducing another ZEP exposure risk for the mammal [66]. To avoid human or domestic animal/pet contact with both racoons and opossums, homeowners should secure and/or remove any food sources outside and ensure their animals are on a leash or in safe housing. This means paying attention to trash cans, dropped fruit, chicken coops, bird feeders, and pet food. It is also important that there are no openings for raccoons or opossums to get inside or under houses, sheds, barns, or other structures [65,66].

When it comes to smaller wild mammals, it is also critical that Florida residents consider how to safely avoid contact with armadillos and squirrels to prevent ZEP transmission. Armadillos like to burrow in soft or sandy soil, which can be in gardens and fields or under homes and other structures [65]. They can create multiple burrows and dens, resulting in significant soil contact. Squirrels, however, prefer to live in trees but feed on the ground [65]. While fallen fruit can attract armadillos, they primarily eat insects (e.g., cockroaches), vegetation, and small invertebrates [65,67]. Squirrels’ diet mostly consists of acorns and other nuts, berries, seeds, and bark [65]. Squirrels often store their food which results in digging in soil, flowerpots, etc. Squirrels, like raccoons and opossums, can also make homes inside of houses and other structures if they have not been properly safeguarded [65]. Squirrels are a source of prey for many other wildlife species throughout Florida.

Finally, bats should also be noted as a synanthropic mammal present in residential areas. While bats prefer a variety of natural habitats depending on each species’ unique needs, they can also utilize houses, barns, garages, and other buildings for roosting [65]. Bats in Florida eat insects, including but not limited to mosquitos, beetles, moths, and more [65]. They are most active at night and depending upon the species, may form colonies [65]. Guano, or bat feces, may be a sign that bats are roosting in an area.

Despite the multiple transmission pathways outlined above, the life cycle of all of the parasites listed involves some kind of fecal exposure. Indiscriminate, untreated, or poorly managed waste from animals and humans is the central driver for the spread of ZEPs found throughout Florida wildlife in this review. It is through infected feces that these parasites contaminate soil, water, insect vectors, food, objects, hands, and hosts leading to infection in susceptible animals and people. Therefore, the best prevention and control efforts to avoid infection with ZEPs remain the removal of untreated waste from our living areas, adherence to safe food preparation and handling practices (including proper cooking temperatures), ensuring that drinking water is safe, washing hands with soap after key events (e.g., animal contact), maintaining the health of pets and livestock through routine veterinary care, and leaving wildlife alone.

Nonetheless, this review is not without its limitations. The results of the search were constrained to our institutional access to select databases and to those publications written in English. Additional publications outside of these restrictions could have yielded more reports of zoonotic enteric parasites in Florida’s synanthropic mammals. Also, the definition for “enteric” became difficult to decipher when considering the complex exposure pathways for several of the included parasites. We chose to incorporate parasites that had a primarily enteric transmission route which were also of public health importance; however, this was not constrained to human gastrointestinal transmission. For example, the zoonotic hookworms included in this review relied on environmental contamination from the infected feces of definitive hosts, but human exposure occurred from direct contact. Future research into this area may choose a more rigid definition. While no included studies were found that examined ZEPs among eastern cottontails or eastern moles of Florida, there could be valuable literature that was simply missed. Additional exploration into the role of these Florida mammals in the risk of zoonotic enteric parasite risks near homes is recommended. And lastly, a risk-of-bias assessment was not conducted for the included studies. This step could have provided additional information related to the integrity or quality of the research cited within this review.

## 5. Conclusions

Each synanthropic mammal included in this review is critical to the health of Florida ecosystems. Safe cohabitation strategies must be implemented to prevent zoonotic enteric parasite exposure to and from wildlife. Limiting or inhibiting residential human–wildlife contact, or wildlife–pet/domestic animal contact, can help to avoid ZEP transmission and proliferation in our shared environment. Utilizing a One Health approach for parasitic disease prevention and control in both people and animals will be the most effective and comprehensive strategy to ensure the health of all.

## Figures and Tables

**Table 1:** Zoonotic enteric parasites in synanthropic mammals of Florida by citation (n = 26).

Category	Parasite	Animal (s)	Reference
Acanthocephala Species	*Acanthocephala* spp.	Coyote	[19]
		Gray Fox	
	*Macracanthorhynchus ingens*	Coyote	[20]
		Racoon	[21,22] *
		Armadillo	[23]
	*Moniliformis clarki*	Squirrel	[24]
	*Moniliformis moniliformis*	Gray Fox	[19]
Cestode Species	*Diphyllobothrium latum*	Coyote	[25]
	*Dipylidium caninum*	Coyote	[25]
	*Hymenolepis* spp.	Coyote	[20,25]
	*Mesocestoides* spp.	Gray Fox	[19]
	*Mesocestoides variabilis*	Racoon	[21,22] *
	*Raillietina bakeri*	Squirrel	[24]
	*Spirometra mansonoides*	Gray Fox	[19]
		Racoon	[21,22,26] *
	*Taenia* spp.	Coyote	[19,25]
		Red Fox	[19]
		Gray Fox	
Nematode Species	*Ancylostoma braziliense* ^^^	Bobcat	[27]
		Gray Fox	[19]
	*Ancylostoma caninum* ^^^	Coyote	[19,20,25]
		Bobcat	[27]
		Red Fox	[19]
		Gray Fox	
	*Angiostrongylus cantonensis*	Armadillo	[28]
	*Angiostrongylus costaricensis*	Racoon	[29]
		Opossum	
	*Ascaris* spp.	Coyote	[25]
	*Baylisascaris procyonis*	Racoon	[30,31]
	*Capillaria aerophila*	Coyote	[19]
		Red Fox	
		Gray Fox	
	*Capillaria plica*	Coyote	[19]
	*Capillaria putorii*	Racoon	[22] *
	*Capillaria* spp.	Racoon	[22] *
		Bat	[32]
	*Gongylonema pulchrum*	Racoon	[22] *
	*Spirurid nematode* spp.	Red Fox	[19]
		Gray Fox	
	*Strongyloides* spp. ^^^	Racoon	[22] *
	*Strongyloides stercoralis* ^^^	Gray Fox	[19]
	*Toxocara canis*	Coyote	[25]
		Red Fox	[19]
	*Trichinella spiralis*	Fox (Undeclared Type)	[18]
		Racoon	[18,22] *
		Opossum	[18]
	*Trichuris vulpis*	Coyote	[19,25]
		Red Fox	[19]
		Gray Fox	
	*Uncinaria stenocephala* ^^^	Coyote	[25]
Protozoa Species	*Blastocystis* sp.	Coyote	[25]
	*Balantidium coli*	Coyote	[25]
	*Cryptosporidium* spp.	Coyote	[25]
	*Cytoisospora* spp. +	Coyote	[25]
	*Entamoeba hystolytica*	Coyote	[25]
	*Trypanosoma cruzi*	Racoon	[33,34,35,36]
		Opossum	[34,35,36,37]
		Armadillo	[36]
		Squirrel	[36]
	*Sarcocystis* spp.	Bobcat	[38]
		Racoon	[39]
	*Toxoplasma gondii*	Bobcat	[40,41]
		Racoon	[40,42]
		Opossum	[40]
		Armadillo	[28,40]
		Squirrel	[40]
Trematode Species	*Alaria* spp.	Coyote	[25]
	*Fibricola cratera*	Racoon	[21,22] *
		Opossum	[43]
	*Heterobilharzia americana* ^^^	Racoon	[20,21,22] *
	*Paragonimus* spp.	Coyote	[25]

* Schaffer et al., 1981 [22] references translocated racoons that originated outside of Florida but were caught and sampled inside the state; ^+^ *Cytoispora* spp. were referred to as *Isopsora* spp. in the original article; ^^^ Zoonotic hookworms and *Heterobilharzia americana* are passed through the feces of definitive hosts but are transmitted to humans via direct contact only. Additional parasites without known or probable zoonotic potential, or gastrointestinal transmission or exposure due to environmental fecal contamination, were excluded from the table.

## Data Availability

No new data were created or analyzed in this study.

## Supplementary Materials

The following supporting information can be downloaded at https://www.mdpi.com/article/10.3390/pathogens13121065/s1, S1: Informal protocol with search strings, dates searched, and results.
