# Supplementary material for: A Systematic Review of Zoonotic Enteric Parasites in Synanthropic Mammalian Species in Florida
Source: Pathogens. 2024 Dec 3;13(12):1065. doi: 10.3390/pathogens13121065 (PMC11728782; doi:10.3390/pathogens13121065)
Supplement: Supplementary file 1 [file pathogens-13-01065-s001.zip › pathogens-3288104-supplementary.pdf]

S1: Informal protocol with the full listing of all search strings used, dates searched, and their corresponding database results.

Pubmed	2022 Results	2024 Results
(armadillo* OR “nine banded armadillo” OR “nine-banded armadillo” OR “long nosed armadillo” OR “long-nosed armadillo” OR “armadillo nueve bandas” OR “dasypus novemcinctus” OR “dasypus novemcinctus aequatorialis” OR “dasypus novemcinctus fenestratus” OR “dasypus novemcinctus hoplites” OR “dasypus novemcinctus mexianae” OR “dasypus novemcinctus mexicanus” OR “dasypus novemcinctus novemcinctus” OR Opossum* OR possum* OR “virginia possum” OR “virginia possums” OR “virginia opossum” OR “virginia opossums” OR “didelphis virginiana” OR raccoon* OR “common raccoon” OR “common racoons” OR “northern raccoon” OR “procyon lotor” OR “procyon lotor auspicatus” OR “procyon lotor elucus” OR “procyon lotor excelsus” OR “procyon lotor fuscipes” OR “procyon lotor gloveralleni” OR “procyon lotor grinnelli” OR “procyon lotor hernandezii” OR “procyon lotor hirtus” OR “procyon lotor incautus” OR “procyon lotor inesperatus” OR “procyon lotor insularis” OR “procyon lotor litoreus” OR “procyon lotor lotor” OR “procyon lotor marinus” OR “procyon lotor maynardi” OR “procyon lotor megalodous” OR “procyon lotor pacificus” OR “procyon lotor pallidus” OR “procyon lotor psora” OR “procyon lotor pumilus” OR “procyon lotor simus” OR “procyon lotor vancouverensis” OR mole OR moles OR “eastern mole” OR “eastern moles” OR “scalopus aquaticus” OR “englewood mole” OR “scalopus aquaticus bassi” OR “scalopus aquaticus anastasae” OR “scalopus aquaticus aquaticus” OR bat OR bats OR “brazilian free-tailed bat” OR “mexican free-tailed bat” OR “tadarida brasiliensis” OR “tadarida brasiliensis Mexicana” OR “tadarida brasiliensis cynocephala” OR “eastern cotton tail” OR “eastern cottontail” OR “florida cottontail” OR “florida cotton tail” OR “sylvilagus floridanus” OR cottontail* OR “gray squirrel” OR “gray squirrels” OR “eastern gray squirrel” OR “eastern gray squirrels” OR “sciurus carolinensis” OR “sciurus carolinensis carolinensis” OR “sciurus carolinensis extimus” OR “sciurus carolinensis fuliginosus” OR “sciurus carolinensis hypophaeus” OR “red fox” OR “red foxes” OR “canis vulpes” OR vulpes OR “gray fox” OR “gray foxes” OR “common gray fox” OR “urocyon cinereoargenteus” OR coyote* OR “canis latrans” OR bobcat* OR “lynx rufus floridanus” OR “wildcat” OR “bay lynx” OR “lynx cat” OR “pallid lynx”) AND ("Zoonotic enteric pathogen" OR "Zoonotic enteric pathogens" OR "Zoonotic enteric parasite" OR "Zoonotic enteric parasites" OR "Zoonotic parasite" OR "Zoonotic parasites" OR "Animal-to-Human parasite" OR "Animal-to-Human parasites" OR "Foodborne parasite" OR "Foodborne parasites" OR "Waterborne parasite" OR "Waterborne parasites" OR "Water-related parasite" OR "Water-related parasites" OR "Enteric parasite" OR "Enteric parasites" OR parasitism OR "Zoonotic intestinal helminth infection" OR "Zoonotic intestinal helminth infections" OR Helminthosis OR Helminthiasis OR Ascarosis OR Ascariasis OR Ancylostomosis OR Ancylostomiasis OR Trichuriosis OR Trichuriasis OR Strongyloidosis OR Strongyloidiasis OR Helminth* OR Ascaris OR Ancylostoma OR Hookworm* OR Trichuris OR Strongyloides OR Alaria OR "rat lungworm" OR "rat lungworms" OR "Echinostoma" OR "Lagochilascaris minor" OR "Zoonotic trypanosomosis" OR Trypanosomiasis OR Chagas OR "Trypanosoma cruzi" OR "Zoonotic intestinal protozoal infection" OR "Zoonotic intestinal protozoal infections" OR Protozoosis OR Protozoasis OR Giardiosis OR Giardiasis OR Cryptosporidiosis OR Blastocystosis OR Sarcocystosis OR Cyclosporiasis OR Cyclospora OR Amoebiasis OR "Amoebic dysentery" OR Entamoeba OR Balantidosis OR Protozoa OR Giardia OR Cryptosporidium OR Blastocystis OR Sarcocystis OR "Cyclospora cayetanensis" OR "Entamoeba histolytica" OR "Balantidium coli" OR Trichinellosis OR Trichinosis OR Trichinella OR Toxoplasmosis OR TORCH OR Toxoplasma OR Toxocarosis OR Toxocariasis OR Toxocariosis OR "Larva migrans" OR Toxocara OR Taeniosis OR Taeniasis OR Tapeworm OR Tapeworms OR Taenia OR "Foodborne trematodosis" OR trematodosis OR Trematodiasis OR Fasciolosis OR Fascioliosis OR Fasciolasis OR Fascioliasis OR Distomatosis OR Fasciolopsiasis OR Fasciolopsiosis OR Opisthorchosis OR Opisthorchiasis OR Clonorchiosis OR Clonorchiasis OR Paragonimos is OR Paragonimiasis OR Metagonimus OR Heterophyiasis OR Fluke OR Flukes OR Trematode OR Trematodes OR Fasciola OR Fasciolopsis OR Opisthorchis OR Clonorchis OR Paragonimus OR "Minute intestinal fluke" OR "Minute intestinal flukes" OR "Haplorchis pumilio" OR "Metagonimus yokogawai" OR "Heterophyes" OR Diphyllobothriosis OR Diphyllobothriasis OR Bothriocephalosis OR Bothriocephaliasis OR Diphyllobothrium OR Bothriocephalus OR "Broad tapeworm" OR "Broad tapeworms" OR "Fish tapeworm" OR "Fish tapeworms" OR Cysticercosis OR Neurocysticercosis OR "Taenia solium" OR "Cystic echinococcosis" OR "Hydatid disease" OR "Hydatid diseases" OR Hydatidosis OR "Echinococcus granulosus" OR "Hydatid cyst" OR "Hydatid cysts" OR "Alveolar echinococcosis" OR "Alveolar hydatidosis" OR "Echinococcus multilocularis") AND (Florid* OR "Southeastern United States”)	238 (10/10)	11 (1/23) *Dates limited to 10/6/22-1/31/24; Same filters

ABI/INFORM Collection	2022 Results	2024 Results
(armadillo* OR “nine banded armadillo” OR “nine-banded armadillo” OR “long nosed armadillo” OR “long-nosed armadillo” OR “armadillo nueve bandas” OR “dasypus novemcinctus” OR “dasypus novemcinctus aequatorialis” OR “dasypus novemcinctus fenestratus” OR “dasypus novemcinctus hoplites” OR “dasypus novemcinctus mexianae” OR “dasypus novemcinctus mexicanus” OR “dasypus novemcinctus novemcinctus” OR Opossum* OR possum* OR “virginia possum” OR “virginia possums” OR “virginia opossum” OR “virginia opossums” OR “didelphis virginiana” OR raccoon* OR “common raccoon” OR “common racoons” OR “northern raccoon” OR “procyon lotor” OR “procyon lotor auspicatus” OR “procyon lotor elucus” OR “procyon lotor excelsus” OR “procyon lotor fuscipes” OR “procyon lotor gloveralleni” OR “procyon lotor grinnelli” OR “procyon lotor hernandezii” OR “procyon lotor hirtus” OR “procyon lotor incautus” OR “procyon lotor inesperatus” OR “procyon lotor insularis” OR “procyon lotor litoreus” OR “procyon lotor lotor” OR “procyon lotor marinus” OR “procyon lotor maynardi” OR “procyon lotor megalodous” OR “procyon lotor pacificus” OR “procyon lotor pallidus” OR “procyon lotor psora” OR “procyon lotor pumilus” OR “procyon lotor simus” OR “procyon lotor vancouverensis” OR mole OR moles OR “eastern mole” OR “eastern moles” OR “scalopus aquaticus” OR “englewood mole” OR “scalopus aquaticus bassi” OR “scalopus aquaticus anastasae” OR “scalopus aquaticus aquaticus” OR bat OR bats OR “brazilian free-tailed bat” OR “mexican free-tailed bat” OR “tadarida brasiliensis” OR “tadarida brasiliensis Mexicana” OR “tadarida brasiliensis cynocephala” OR “eastern cotton tail” OR “eastern cottontail” OR “florida cottontail” OR “florida cotton tail” OR “sylvilagus floridanus” OR cottontail* OR “gray squirrel” OR “gray squirrels” OR “eastern gray squirrel” OR “eastern gray squirrels” OR “sciurus carolinensis” OR “sciurus carolinensis carolinensis” OR “sciurus carolinensis extimus” OR “sciurus carolinensis fuliginosus” OR “sciurus carolinensis hypophaeus” OR “red fox” OR “red foxes” OR “canis vulpes” OR vulpes OR “gray fox” OR “gray foxes” OR “common gray fox” OR “urocyon cinereoargenteus” OR coyote* OR “canis latrans” OR bobcat* OR “lynx rufus floridanus” OR “wildcat” OR “bay lynx” OR “lynx cat” OR “pallid lynx”) AND ("Zoonotic enteric pathogen" OR "Zoonotic enteric pathogens" OR "Zoonotic enteric parasite" OR "Zoonotic enteric parasites" OR "Zoonotic parasite" OR "Zoonotic parasites" OR "Animal-to-Human parasite" OR "Animal-to-Human parasites" OR "Foodborne parasite" OR "Foodborne parasites" OR "Waterborne parasite" OR "Waterborne parasites" OR "Water-related parasite" OR "Water-related parasites" OR "Enteric parasite" OR "Enteric parasites" OR parasitism OR "Zoonotic intestinal helminth infection" OR "Zoonotic intestinal helminth infections" OR Helminthosis OR Helminthiasis OR Ascarosis OR Ascariasis OR Ancylostomosis OR Ancylostomiasis OR Trichuriosis OR Trichuriasis OR Strongyloidosis OR Strongyloidiasis OR Helminth* OR Ascaris OR Ancylostoma OR Hookworm* OR Trichuris OR Strongyloides OR Alaria OR "rat lungworm" OR "rat lungworms" OR "Echinostoma" OR "Lagochilascaris minor" OR "Zoonotic trypanosomosis" OR Trypanosomiasis OR Chagas OR "Trypanosoma cruzi" OR "Zoonotic intestinal protozoal infection" OR "Zoonotic intestinal protozoal infections" OR Protozoosis OR Protozoasis OR Giardiosis OR Giardiasis OR Cryptosporidiosis OR Blastocystosis OR Sarcocystosis OR Cyclosporiasis OR Cyclospora OR Amoebiasis OR "Amoebic dysentery" OR Entamoeba OR Balantidosis OR Protozoa OR Giardia OR Cryptosporidium OR Blastocystis OR Sarcocystis OR "Cyclospora cayetanensis" OR "Entamoeba histolytica" OR "Balantidium coli" OR Trichinellosis OR Trichinosis OR Trichinella OR Toxoplasmosis OR TORCH OR Toxoplasma OR Toxocarosis OR Toxocariasis OR Toxocariosis OR "Larva migrans" OR Toxocara OR Taeniosis OR Taeniasis OR Tapeworm OR Tapeworms OR Taenia OR "Foodborne trematodosis" OR trematodosis OR Trematodiasis OR Fasciolosis OR Fascioliosis OR Fasciolasis OR Fascioliasis OR Distomatosis OR Fasciolopsiasis OR Fasciolopsiosis OR Opisthorchosis OR Opisthorchiasis OR Clonorchiosis OR Clonorchiasis OR Paragonimos is OR Paragonimiasis OR Metagonimus OR Heterophyiasis OR Fluke OR Flukes OR Trematode OR Trematodes OR Fasciola OR Fasciolopsis OR Opisthorchis OR Clonorchis OR Paragonimus OR "Minute intestinal fluke" OR "Minute intestinal flukes" OR "Haplorchis pumilio" OR "Metagonimus yokogawai" OR "Heterophyes" OR Diphyllobothriosis OR Diphyllobothriasis OR Bothriocephalosis OR Bothriocephaliasis OR Diphyllobothrium OR Bothriocephalus OR "Broad tapeworm" OR "Broad tapeworms" OR "Fish tapeworm" OR "Fish tapeworms" OR Cysticercosis OR Neurocysticercosis OR "Taenia solium" OR "Cystic echinococcosis" OR "Hydatid disease" OR "Hydatid diseases" OR Hydatidosis OR "Echinococcus granulosus" OR "Hydatid cyst" OR "Hydatid cysts" OR "Alveolar echinococcosis" OR "Alveolar hydatidosis" OR "Echinococcus multilocularis") AND (Florid* OR "Southeastern United States”)<br><br>**FILTERS:** Scholarly journals	99 (10/6)	10 (1/16) *Dates limited to 10/6/22-1/31/24; Same filters
**Agricola**	**2022 Results**	**2024 Results**

(armadillo* OR “nine banded armadillo” OR “nine-banded armadillo” OR “long nosed armadillo” OR “long-nosed armadillo” OR “armadillo nueve bandas” OR “dasypus novemcinctus” OR “dasypus novemcinctus aequatorialis” OR “dasypus novemcinctus fenestratus” OR “dasypus novemcinctus hoplites” OR “dasypus novemcinctus mexianae” OR “dasypus novemcinctus mexicanus” OR “dasypus novemcinctus novemcinctus” OR Opossum* OR possum* OR “virginia possum” OR “virginia possums” OR “virginia opossum” OR “virginia opossums” OR “didelphis virginiana” OR raccoon* OR “common raccoon” OR “common racoons” OR “northern raccoon” OR “procyon lotor” OR “procyon lotor auspicatus” OR “procyon lotor elucus” OR “procyon lotor excelsus” OR “procyon lotor fuscipes” OR “procyon lotor gloveralleni” OR “procyon lotor grinnelli” OR “procyon lotor hernandezii” OR “procyon lotor hirtus” OR “procyon lotor incautus” OR “procyon lotor inesperatus” OR “procyon lotor insularis” OR “procyon lotor litoreus” OR “procyon lotor lotor” OR “procyon lotor marinus” OR “procyon lotor maynardi” OR “procyon lotor megalodous” OR “procyon lotor pacificus” OR “procyon lotor pallidus” OR “procyon lotor psora” OR “procyon lotor pumilus” OR “procyon lotor simus” OR “procyon lotor vancouverensis” OR mole OR moles OR “eastern mole” OR “eastern moles” OR “scalopus aquaticus” OR “englewood mole” OR “scalopus aquaticus bassi” OR “scalopus aquaticus anastasae” OR “scalopus aquaticus aquaticus” OR bat OR bats OR “brazilian free-tailed bat” OR “mexican free-tailed bat” OR “tadarida brasiliensis” OR “tadarida brasiliensis Mexicana” OR “tadarida brasiliensis cynocephala” OR “eastern cotton tail” OR “eastern cottontail” OR “florida cottontail” OR “florida cotton tail” OR “sylvilagus floridanus” OR cottontail* OR “gray squirrel” OR “gray squirrels” OR “eastern gray squirrel” OR “eastern gray squirrels” OR “sciurus carolinensis” OR “sciurus carolinensis carolinensis” OR “sciurus carolinensis extimus” OR “sciurus carolinensis fuliginosus” OR “sciurus carolinensis hypophaeus” OR “red fox” OR “red foxes” OR “canis vulpes” OR vulpes OR “gray fox” OR “gray foxes” OR “common gray fox” OR “urocyon cinereoargenteus” OR coyote* OR “canis latrans” OR bobcat* OR “lynx rufus floridanus” OR “wildcat” OR “bay lynx” OR “lynx cat” OR “pallid lynx”) AND ("Zoonotic enteric pathogen" OR "Zoonotic enteric pathogens" OR "Zoonotic enteric parasite" OR "Zoonotic enteric parasites" OR "Zoonotic parasite" OR "Zoonotic parasites" OR "Animal-to-Human parasite" OR "Animal-to-Human parasites" OR "Foodborne parasite" OR "Foodborne parasites" OR "Waterborne parasite" OR "Waterborne parasites" OR "Water-related parasite" OR "Water-related parasites" OR "Enteric parasite" OR "Enteric parasites" OR parasitism OR "Zoonotic intestinal helminth infection" OR "Zoonotic intestinal helminth infections" OR Helminthosis OR Helminthiasis OR Ascarosis OR Ascariasis OR Ancylostomosis OR Ancylostomiasis OR Trichuriosis OR Trichuriasis OR Strongyloidosis OR Strongyloidiasis OR Helminth* OR Ascaris OR Ancylostoma OR Hookworm* OR Trichuris OR Strongyloides OR Alaria OR "rat lungworm" OR "rat lungworms" OR "Echinostoma" OR "Lagochilascaris minor" OR "Zoonotic trypanosomosis" OR Trypanosomiasis OR Chagas OR "Trypanosoma cruzi" OR "Zoonotic intestinal protozoal infection" OR "Zoonotic intestinal protozoal infections" OR Protozoosis OR Protozoasis OR Giardiosis OR Giardiasis OR Cryptosporidiosis OR Blastocystosis OR Sarcocystosis OR Cyclosporiasis OR Cyclospora OR Amoebiasis OR "Amoebic dysentery" OR Entamoeba OR Balantidosis OR Protozoa OR Giardia OR Cryptosporidium OR Blastocystis OR Sarcocystis OR "Cyclospora cayetanensis" OR "Entamoeba histolytica" OR "Balantidium coli" OR Trichinellosis OR Trichinosis OR Trichinella OR Toxoplasmosis OR TORCH OR Toxoplasma OR Toxocarosis OR Toxocariasis OR Toxocariosis OR "Larva migrans" OR Toxocara OR Taeniosis OR Taeniasis OR Tapeworm OR Tapeworms OR Taenia OR "Foodborne trematodosis" OR trematodosis OR Trematodiasis OR Fasciolosis OR Fascioliosis OR Fasciolasis OR Fascioliasis OR Distomatosis OR Fasciolopsiasis OR Fasciolopsiosis OR Opisthorchosis OR Opisthorchiasis OR Clonorchiosis OR Clonorchiasis OR Paragonimos is OR Paragonimiasis OR Metagonimus OR Heterophyiasis OR Fluke OR Flukes OR Trematode OR Trematodes OR Fasciola OR Fasciolopsis OR Opisthorchis OR Clonorchis OR Paragonimus OR "Minute intestinal fluke" OR "Minute intestinal flukes" OR "Haplorchis pumilio" OR "Metagonimus yokogawai" OR "Heterophyes" OR Diphyllobothriosis OR Diphyllobothriasis OR Bothriocephalosis OR Bothriocephaliasis OR Diphyllobothrium OR Bothriocephalus OR "Broad tapeworm" OR "Broad tapeworms" OR "Fish tapeworm" OR "Fish tapeworms" OR Cysticercosis OR Neurocysticercosis OR "Taenia solium" OR "Cystic echinococcosis" OR "Hydatid disease" OR "Hydatid diseases" OR Hydatidosis OR "Echinococcus granulosus" OR "Hydatid cyst" OR "Hydatid cysts" OR "Alveolar echinococcosis" OR "Alveolar hydatidosis" OR "Echinococcus multilocularis") AND (Florid* OR "Southeastern United States”)<br><br>**FILTERS:** Scholarly journals	52<br>10/9	1<br>(1/16)<br>*Dates limited to 10/9/22-1/31/24; Same filters
**Agriculture Collection**	**2022 Results**	**2024 Results**
Keyword: "mammal" AND<br>Keyword: "parasite" OR<br>Keyword: “zoonotic diseases” OR	1,239<br>(10/10)	144<br>(1/16)

Keyword: “zoonotic parasites” AND<br>Keyword: florid* OR<br>Keyword: "southeastern united states"<br><br>**FILTERS:** Sorted by: Academic Journals; Peer-Reviewed journals		*Dates limited to 10/10/22-1/31/24; Same filters
**Aquatic Sciences Collection**	**2022 Results**	**2024 Results**
(armadillo* OR “nine banded armadillo” OR “nine-banded armadillo” OR “long nosed armadillo” OR “long-nosed armadillo” OR “armadillo nueve bandas” OR “dasypus novemcinctus” OR “dasypus novemcinctus aequatorialis” OR “dasypus novemcinctus fenestratus” OR “dasypus novemcinctus hoplites” OR “dasypus novemcinctus mexianae” OR “dasypus novemcinctus mexicanus” OR “dasypus novemcinctus novemcinctus” OR Opossum* OR possum* OR “virginia possum” OR “virginia possums” OR “virginia opossum” OR “virginia opossums” OR “didelphis virginiana” OR raccoon* OR “common raccoon” OR “common racoons” OR “northern raccoon” OR “procyon lotor” OR “procyon lotor auspicatus” OR “procyon lotor elucus” OR “procyon lotor excelsus” OR “procyon lotor fuscipes” OR “procyon lotor gloveralleni” OR “procyon lotor grinnelli” OR “procyon lotor hernandezii” OR “procyon lotor hirtus” OR “procyon lotor incautus” OR “procyon lotor inesperatus” OR “procyon lotor insularis” OR “procyon lotor litoreus” OR “procyon lotor lotor” OR “procyon lotor marinus” OR “procyon lotor maynardi” OR “procyon lotor megalodous” OR “procyon lotor pacificus” OR “procyon lotor pallidus” OR “procyon lotor psora” OR “procyon lotor pumilus” OR “procyon lotor simus” OR “procyon lotor vancouverensis” OR mole OR moles OR “eastern mole” OR “eastern moles” OR “scalopus aquaticus” OR “englewood mole” OR “scalopus aquaticus bassi” OR “scalopus aquaticus anastasae” OR “scalopus aquaticus aquaticus” OR bat OR bats OR “brazilian free-tailed bat” OR “mexican free-tailed bat” OR “tadarida brasiliensis” OR “tadarida brasiliensis Mexicana” OR “tadarida brasiliensis cynocephala” OR “eastern cotton tail” OR “eastern cottontail” OR “florida cottontail” OR “florida cotton tail” OR “sylvilagus floridanus” OR cottontail* OR “gray squirrel” OR “gray squirrels” OR “eastern gray squirrel” OR “eastern gray squirrels” OR “sciurus carolinensis” OR “sciurus carolinensis carolinensis” OR “sciurus carolinensis extimus” OR “sciurus carolinensis fuliginosus” OR “sciurus carolinensis hypophaeus” OR “red fox” OR “red foxes” OR “canis vulpes” OR vulpes OR “gray fox” OR “gray foxes” OR “common gray fox” OR “urocyon cinereoargenteus” OR coyote* OR “canis latrans” OR bobcat* OR “lynx rufus floridanus” OR “wildcat” OR “bay lynx” OR “lynx cat” OR “pallid lynx”) AND ("Zoonotic enteric pathogen" OR "Zoonotic enteric pathogens" OR "Zoonotic enteric parasite" OR "Zoonotic enteric parasites" OR "Zoonotic parasite" OR "Zoonotic parasites" OR "Animal-to-Human parasite" OR "Animal-to-Human parasites" OR "Foodborne parasite" OR "Foodborne parasites" OR "Waterborne parasite" OR "Waterborne parasites" OR "Water-related parasite" OR "Water-related parasites" OR "Enteric parasite" OR "Enteric parasites" OR parasitism OR "Zoonotic intestinal helminth infection" OR "Zoonotic intestinal helminth infections" OR Helminthosis OR Helminthiasis OR Ascarosis OR Ascariasis OR Ancylostomosis OR Ancylostomiasis OR Trichuriosis OR Trichuriasis OR Strongyloidosis OR Strongyloidiasis OR Helminth* OR Ascaris OR Ancylostoma OR Hookworm* OR Trichuris OR Strongyloides OR Alaria OR "rat lungworm" OR "rat lungworms" OR "Echinostoma" OR "Lagochilascaris minor" OR "Zoonotic trypanosomosis" OR Trypanosomiasis OR Chagas OR "Trypanosoma cruzi" OR "Zoonotic intestinal protozoal infection" OR "Zoonotic intestinal protozoal infections" OR Protozoosis OR Protozoasis OR Giardiosis OR Giardiasis OR Cryptosporidiosis OR Blastocystosis OR Sarcocystosis OR Cyclosporiasis OR Cyclospora OR Amoebiasis OR "Amoebic dysentery" OR Entamoeba OR Balantidosis OR Protozoa OR Giardia OR Cryptosporidium OR Blastocystis OR Sarcocystis OR "Cyclospora cayetanensis" OR "Entamoeba histolytica" OR "Balantidium coli" OR Trichinellosis OR Trichinosis OR Trichinella OR Toxoplasmosis OR TORCH OR Toxoplasma OR Toxocarosis OR Toxocariasis OR Toxocariosis OR "Larva migrans" OR Toxocara OR Taeniosis OR Taeniasis OR Tapeworm OR Tapeworms OR Taenia OR "Foodborne trematodosis" OR trematodosis OR Trematodiasis OR Fasciolosis OR Fascioliosis OR Fasciolasis OR Fascioliasis OR Distomatosis OR Fasciolopsiasis OR Fasciolopsiosis OR Opisthorchosis OR Opisthorchiasis OR Clonorchiosis OR Clonorchiasis OR Paragonimosis OR Paragonimiasis OR Metagonimus OR Heterophyiasis OR Fluke OR Flukes OR Trematode OR Trematodes OR Fasciola OR Fasciolopsis OR Opisthorchis OR Clonorchis OR Paragonimus OR "Minute intestinal fluke" OR "Minute intestinal flukes" OR "Haplorchis pumilio" OR "Metagonimus yokogawai" OR "Heterophyes" OR Diphyllobothriosis OR Diphyllobothriasis OR Bothriocephalosis OR Bothriocephaliasis OR Diphyllobothrium OR Bothriocephalus OR "Broad tapeworm" OR "Broad tapeworms" OR "Fish tapeworm" OR "Fish tapeworms" OR Cysticercosis OR Neurocysticercosis OR "Taenia solium" OR "Cystic echinococcosis" OR "Hydatid disease" OR "Hydatid diseases" OR Hydatidosis OR	851 (10/11)	51 (1/16) *Dates limited to 10/11/22-1/31/24; Same filters

"Echinococcus granulosus" OR "Hydatid cyst" OR "Hydatid cysts" OR "Alveolar echinococcosis" OR "Alveolar hydatidosis" OR "Echinococcus multilocularis") AND (Florid* OR "Southeastern United States")<br><br>**FILTERS:** Scholarly journals only		
**Biological Sciences**	**2022 Results**	**2024 Results**
(armadillo* OR "nine banded armadillo" OR "nine-banded armadillo" OR "long nosed armadillo" OR "long-nosed armadillo" OR "armadillo nueve bandas" OR "dasypus novemcinctus" OR "dasypus novemcinctus aequatorialis" OR "dasypus novemcinctus fenestratus" OR "dasypus novemcinctus hoplites" OR "dasypus novemcinctus mexianae" OR "dasypus novemcinctus mexicanus" OR "dasypus novemcinctus novemcinctus" OR Opossum* OR possum* OR "virginia possum" OR "virginia possums" OR "virginia opossum" OR "virginia opossums" OR "didelphis virginiana" OR raccoon* OR "common raccoon" OR "common racoons" OR "northern raccoon" OR "procyon lotor" OR "procyon lotor auspicatus" OR "procyon lotor elucus" OR "procyon lotor excelsus" OR "procyon lotor fuscipes" OR "procyon lotor gloveralleni" OR "procyon lotor grinnelli" OR "procyon lotor hernandezii" OR "procyon lotor hirtus" OR "procyon lotor incautus" OR "procyon lotor inesperatus" OR "procyon lotor insularis" OR "procyon lotor litoreus" OR "procyon lotor lotor" OR "procyon lotor marinus" OR "procyon lotor maynardi" OR "procyon lotor megalodous" OR "procyon lotor pacificus" OR "procyon lotor pallidus" OR "procyon lotor psora" OR "procyon lotor pumilus" OR "procyon lotor simus" OR "procyon lotor vancouverensis" OR mole OR moles OR "eastern mole" OR "eastern moles" OR "scalopus aquaticus" OR "englewood mole" OR "scalopus aquaticus bassi" OR "scalopus aquaticus anastasae" OR "scalopus aquaticus aquaticus" OR bat OR bats OR "brazilian free-tailed bat" OR "mexican free-tailed bat" OR "tadarida brasiliensis" OR "tadarida brasiliensis Mexicana" OR "tadarida brasiliensis cynocephala" OR "eastern cotton tail" OR "eastern cottontail" OR "florida cottontail" OR "florida cotton tail" OR "sylvilagus floridanus" OR cottontail* OR "gray squirrel" OR "gray squirrels" OR "eastern gray squirrel" OR "eastern gray squirrels" OR "sciurus carolinensis" OR "sciurus carolinensis carolinensis" OR "sciurus carolinensis extimus" OR "sciurus carolinensis fuliginosus" OR "sciurus carolinensis hypophaeus" OR "red fox" OR "red foxes" OR "canis vulpes" OR vulpes OR "gray fox" OR "gray foxes" OR "common gray fox" OR "urocyon cinereoargenteus" OR coyote* OR "canis latrans" OR bobcat* OR "lynx rufus floridanus" OR "wildcat" OR "bay lynx" OR "lynx cat" OR "pallid lynx") AND ("Zoonotic enteric pathogen" OR "Zoonotic enteric pathogens" OR "Zoonotic enteric parasite" OR "Zoonotic enteric parasites" OR "Zoonotic parasite" OR "Zoonotic parasites" OR "Animal-to-Human parasite" OR "Animal-to-Human parasites" OR "Foodborne parasite" OR "Foodborne parasites" OR "Waterborne parasite" OR "Waterborne parasites" OR "Water-related parasite" OR "Water-related parasites" OR "Enteric parasite" OR "Enteric parasites" OR parasitism OR "Zoonotic intestinal helminth infection" OR "Zoonotic intestinal helminth infections" OR Helminthosis OR Helminthiasis OR Ascarosis OR Ascariasis OR Ancylostomosis OR Ancylostomiasis OR Trichuriosis OR Trichuriasis OR Strongyloidosis OR Strongyloidiasis OR Helminth* OR Ascaris OR Ancylostoma OR Hookworm* OR Trichuris OR Strongyloides OR Alaria OR "rat lungworm" OR "rat lungworms" OR "Echinostoma" OR "Lagochilascaris minor" OR "Zoonotic trypanosomosis" OR Trypanosomiasis OR Chagas OR "Trypanosoma cruzi" OR "Zoonotic intestinal protozoal infection" OR "Zoonotic intestinal protozoal infections" OR Protozoosis OR Protozoasis OR Giardiosis OR Giardiasis OR Cryptosporidiosis OR Blastocystosis OR Sarcocystosis OR Cyclosporiasis OR Cyclospora OR Amoebiasis OR "Amoebic dysentery" OR Entamoeba OR Balantidosis OR Protozoa OR Giardia OR Cryptosporidium OR Blastocystis OR Sarcocystis OR "Cyclospora cayetanensis" OR "Entamoeba histolytica" OR "Balantidium coli" OR Trichinellosis OR Trichinosis OR Trichinella OR Toxoplasmosis OR TORCH OR Toxoplasma OR Toxocarosis OR Toxocariasis OR Toxocariosis OR "Larva migrans" OR Toxocara OR Taeniosis OR Taeniasis OR Tapeworm OR Tapeworms OR Taenia OR "Foodborne trematodosis" OR trematodosis OR Trematodiasis OR Fasciolosis OR Fascioliosis OR Fasciolasis OR Fascioliasis OR Distomatosis OR Fasciolopsiasis OR Fasciolopsiosis OR Opisthorchosis OR Opisthorchiasis OR Clonorchiosis OR Clonorchiasis OR Paragonimosis OR Paragonimiasis OR Metagonimus OR Heterophyiasis OR Fluke OR Flukes OR Trematode OR Trematodes OR Fasciola OR Fasciolopsis OR Opisthorchis OR Clonorchis OR Paragonimus OR "Minute intestinal fluke" OR "Minute intestinal flukes" OR "Haplorchis pumilio" OR "Metagonimus yokogawai" OR "Heterophyes" OR Diphyllobothriosis OR Diphyllobothriasis OR Bothriocephalosis OR Bothriocephaliasis OR Diphyllobothrium OR Bothriocephalus OR "Broad tapeworm" OR "Broad tapeworms" OR "Fish tapeworm" OR "Fish tapeworms" OR Cysticercosis OR Neurocysticercosis OR "Taenia solium" OR "Cystic echinococcosis" OR "Hydatid disease" OR "Hydatid diseases" OR Hydatidosis OR "Echinococcus granulosus" OR "Hydatid cyst" OR "Hydatid cysts" OR "Alveolar echinococcosis" OR "Alveolar hydatidosis" OR "Echinococcus multilocularis") AND (Florid* OR "Southeastern United States")	1,938 (10/11)	159 (1/23) *Dates limited to 10/11/22-1/31/24; Same filters

**FILTERS:** Scholarly journals only; Peer-reviewed only		
**BioOne Complete**	**2022 Results**	**2024 Results**
Keyword: "mammal" AND<br>Keyword: "parasite" OR<br>Keyword: "zoonotic disease" OR<br>Keyword: "zoonotic parasites" AND<br>Keyword: florid* OR<br>Keyword: "southeastern united states"<br><br>**FILTERS:** (none)	0 (10/6)	0 (1/23)
**Environment Complete**	**2022 Results**	**2024 Results**
AB((armadillo* OR "nine banded armadillo" OR "nine-banded armadillo" OR "long nosed armadillo" OR "long-nosed armadillo" OR "armadillo nueve bandas" OR "dasypus novemcinctus" OR "dasypus novemcinctus aequatorialis" OR "dasypus novemcinctus fenestratus" OR "dasypus novemcinctus hoplites" OR "dasypus novemcinctus mexianae" OR "dasypus novemcinctus mexicanus" OR "dasypus novemcinctus novemcinctus" OR Opossum* OR possum* OR "virginia possum" OR "virginia possums" OR "virginia opossum" OR "virginia opossums" OR "didelphis virginiana" OR raccoon* OR "common raccoon" OR "common racoons" OR "northern raccoon" OR "procyon lotor" OR "procyon lotor auspicatus" OR "procyon lotor elucus" OR "procyon lotor excelsus" OR "procyon lotor fuscipes" OR "procyon lotor gloveralleni" OR "procyon lotor grinnelli" OR "procyon lotor hernandezii" OR "procyon lotor hirtus" OR "procyon lotor incautus" OR "procyon lotor inesperatus" OR "procyon lotor insularis" OR "procyon lotor litoreus" OR "procyon lotor lotor" OR "procyon lotor marinus" OR "procyon lotor maynardi" OR "procyon lotor megalodous" OR "procyon lotor pacificus" OR "procyon lotor pallidus" OR "procyon lotor psora" OR "procyon lotor pumilus" OR "procyon lotor simus" OR "procyon lotor vancouverensis" OR mole OR moles OR "eastern mole" OR "eastern moles" OR "scalopus aquaticus" OR "englewood mole" OR "scalopus aquaticus bassi" OR "scalopus aquaticus anastasae" OR "scalopus aquaticus aquaticus" OR bat OR bats OR "brazilian free-tailed bat" OR "mexican free-tailed bat" OR "tadarida brasiliensis" OR "tadarida brasiliensis Mexicana" OR "tadarida brasiliensis cynocephala" OR "eastern cotton tail" OR "eastern cottontail" OR "florida cottontail" OR "florida cotton tail" OR "sylvilagus floridanus" OR cottontail* OR "gray squirrel" OR "gray squirrels" OR "eastern gray squirrel" OR "eastern gray squirrels" OR "sciurus carolinensis" OR "sciurus carolinensis carolinensis" OR "sciurus carolinensis extimus" OR "sciurus carolinensis fuliginosus" OR "sciurus carolinensis hypophaeus" OR "red fox" OR "red foxes" OR "canis vulpes" OR vulpes OR "gray fox" OR "gray foxes" OR "common gray fox" OR "urocyon cinereoargenteus" OR coyote* OR "canis latrans" OR bobcat* OR "lynx rufus floridanus" OR "wildcat" OR "bay lynx" OR "lynx cat" OR "pallid lynx") AND ("Zoonotic enteric pathogen" OR "Zoonotic enteric pathogens" OR "Zoonotic enteric parasite" OR "Zoonotic enteric parasites" OR "Zoonotic parasite" OR "Zoonotic parasites" OR "Animal-to-Human parasite" OR "Animal-to-Human parasites" OR "Foodborne parasite" OR "Foodborne parasites" OR "Waterborne parasite" OR "Waterborne parasites" OR "Water-related parasite" OR "Water-related parasites" OR "Enteric parasite" OR "Enteric parasites" OR parasitism OR "Zoonotic intestinal helminth infection" OR "Zoonotic intestinal helminth infections" OR Helminthosis OR Helminthiasis OR Ascarosis OR Ascariasis OR Ancylostomosis OR Ancylostomiasis OR Trichuriosis OR Trichuriasis OR Strongyloidosis OR Strongyloidiasis OR Helminth* OR Ascaris OR Ancylostoma OR Hookworm* OR Trichuris OR Strongyloides OR Alaria OR "rat lungworm" OR "rat lungworms" OR "Echinostoma" OR "Lagochilascaris minor" OR "Zoonotic trypanosomosis" OR Trypanosomiasis OR Chagas OR "Trypanosoma cruzi" OR "Zoonotic intestinal protozoal infection" OR "Zoonotic intestinal protozoal infections" OR Protozoosis OR Protozoasis OR Giardiosis OR Giardiasis OR Cryptosporidiosis OR Blastocystosis OR Sarcocystosis OR Cyclosporiasis OR Cyclospora OR Amoebiasis OR "Amoebic dysentery" OR Entamoeba OR Balantidosis OR Protozoa OR Giardia OR Cryptosporidium OR Blastocystis OR Sarcocystis OR "Cyclospora cayetanensis" OR "Entamoeba histolytica" OR "Balantidium coli" OR Trichinellosis OR Trichinosis OR Trichinella OR Toxoplasmosis OR TORCH OR Toxoplasma OR Toxocarosis OR Toxocariasis OR Toxocariosis OR "Larva migrans" OR Toxocara OR Taeniosis OR Taeniasis OR Tapeworm OR Tapeworms OR Taenia OR "Foodborne trematodosis" OR trematodosis OR Trematodiasis OR Fasciolosis OR Fascioliosis OR Fasciolasis OR Fascioliasis OR Distomatosis OR Fasciolopsiasis OR Fasciolopsiosis OR Opisthorchosis OR Opisthorchiasis OR Clonorchiosis OR Clonorchiasis OR Paragonimos is OR Paragonimiasis OR Metagonimus OR Heterophyiasis OR Fluke OR Flukes OR Trematode OR Trematodes OR Fasciola OR Fasciolopsis OR	8 (10/6/22)	3 (1/23) *Same filters

Opisthorchis OR Clonorchis OR Paragonimus OR "Minute intestinal fluke" OR "Minute intestinal flukes" OR "Haplorchis pumilio" OR "Metagonimus yokogawai" OR "Heterophyes" OR Diphyllobothriosis OR Diphyllobothriasis OR Bothriocephalosis OR Bothriocephaliasis OR Diphyllobothrium OR Bothriocephalus OR "Broad tapeworm" OR "Broad tapeworms" OR "Fish tapeworm" OR "Fish tapeworms" OR Cysticercosis OR Neurocysticercosis OR "Taenia solium" OR "Cystic echinococcosis" OR "Hydatid disease" OR "Hydatid diseases" OR Hydatidosis OR "Echinococcus granulosus" OR "Hydatid cyst" OR "Hydatid cysts" OR "Alveolar echinococcosis" OR "Alveolar hydatidosis" OR "Echinococcus multilocularis") AND (Florid* OR "Southeastern United States")) <br>**FILTERS:** Academic journals only		
**Agricultural and Environmental Science Collection**	**2022 Results**	**2024 Results**
**AB,TI**((armadillo* OR "nine banded armadillo" OR "nine-banded armadillo" OR "long nosed armadillo" OR "long-nosed armadillo" OR "armadillo nueve bandas" OR "dasypus novemcinctus" OR "dasypus novemcinctus aequatorialis" OR "dasypus novemcinctus fenestratus" OR "dasypus novemcinctus hoplites" OR "dasypus novemcinctus mexianae" OR "dasypus novemcinctus mexicanus" OR "dasypus novemcinctus novemcinctus" OR Opossum* OR possum* OR "virginia possum" OR "virginia possums" OR "virginia opossum" OR "virginia opossums" OR "didelphis virginiana" OR raccoon* OR "common raccoon" OR "common racoons" OR "northern raccoon" OR "procyon lotor" OR "procyon lotor auspicatus" OR "procyon lotor elucus" OR "procyon lotor excelsus" OR "procyon lotor fuscipes" OR "procyon lotor gloveralleni" OR "procyon lotor grinnelli" OR "procyon lotor hernandezii" OR "procyon lotor hirtus" OR "procyon lotor incautus" OR "procyon lotor inesperatus" OR "procyon lotor insularis" OR "procyon lotor litoreus" OR "procyon lotor lotor" OR "procyon lotor marinus" OR "procyon lotor maynardi" OR "procyon lotor megalodous" OR "procyon lotor pacificus" OR "procyon lotor pallidus" OR "procyon lotor psora" OR "procyon lotor pumilus" OR "procyon lotor simus" OR "procyon lotor vancouverensis" OR mole OR moles OR "eastern mole" OR "eastern moles" OR "scalopus aquaticus" OR "englewood mole" OR "scalopus aquaticus bassi" OR "scalopus aquaticus anastasae" OR "scalopus aquaticus aquaticus" OR bat OR bats OR "brazilian free-tailed bat" OR "mexican free-tailed bat" OR "tadarida brasiliensis" OR "tadarida brasiliensis Mexicana" OR "tadarida brasiliensis cynocephala" OR "eastern cotton tail" OR "eastern cottontail" OR "florida cottontail" OR "florida cotton tail" OR "sylvilagus floridanus" OR cottontail* OR "gray squirrel" OR "gray squirrels" OR "eastern gray squirrel" OR "eastern gray squirrels" OR "sciurus carolinensis" OR "sciurus carolinensis carolinensis" OR "sciurus carolinensis extimus" OR "sciurus carolinensis fuliginosus" OR "sciurus carolinensis hypophaeus" OR "red fox" OR "red foxes" OR "canis vulpes" OR vulpes OR "gray fox" OR "gray foxes" OR "common gray fox" OR "urocyon cinereoargenteus" OR coyote* OR "canis latrans" OR bobcat* OR "lynx rufus floridanus" OR "wildcat" OR "bay lynx" OR "lynx cat" OR "pallid lynx") AND ("Zoonotic enteric pathogen" OR "Zoonotic enteric pathogens" OR "Zoonotic enteric parasite" OR "Zoonotic enteric parasites" OR "Zoonotic parasite" OR "Zoonotic parasites" OR "Animal-to-Human parasite" OR "Animal-to-Human parasites" OR "Foodborne parasite" OR "Foodborne parasites" OR "Waterborne parasite" OR "Waterborne parasites" OR "Water-related parasite" OR "Water-related parasites" OR "Enteric parasite" OR "Enteric parasites" OR parasitism OR "Zoonotic intestinal helminth infection" OR "Zoonotic intestinal helminth infections" OR Helminthosis OR Helminthiasis OR Ascarosis OR Ascariasis OR Ancylostomosis OR Ancylostomiasis OR Trichuriosis OR Trichuriasis OR Strongyloidosis OR Strongyloidiasis OR Helminth* OR Ascaris OR Ancylostoma OR Hookworm* OR Trichuris OR Strongyloides OR Alaria OR "rat lungworm" OR "rat lungworms" OR "Echinostoma" OR "Lagochilascaris minor" OR "Zoonotic trypanosomosis" OR Trypanosomiasis OR Chagas OR "Trypanosoma cruzi" OR "Zoonotic intestinal protozoal infection" OR "Zoonotic intestinal protozoal infections" OR Protozoosis OR Protozoasis OR Giardiosis OR Giardiasis OR Cryptosporidiosis OR Blastocystosis OR Sarcocystosis OR Cyclosporiasis OR Cyclospora OR Amoebiasis OR "Amoebic dysentery" OR Entamoeba OR Balantidosis OR Protozoa OR Giardia OR Cryptosporidium OR Blastocystis OR Sarcocystis OR "Cyclospora cayetanensis" OR "Entamoeba histolytica" OR "Balantidium coli" OR Trichinellosis OR Trichinosis OR Trichinella OR Toxoplasmosis OR TORCH OR Toxoplasma OR Toxocarosis OR Toxocariasis OR Toxocariosis OR "Larva migrans" OR Toxocara OR Taeniosis OR Taeniasis OR Tapeworm OR Tapeworms OR Taenia OR "Foodborne trematodosis" OR trematodosis OR Trematodiasis OR Fasciolosis OR Fascioliosis OR Fasciolasis OR Fascioliasis OR Distomatosis OR Fasciolopsiasis OR Fasciolopsiosis OR Opisthorchosis OR Opisthorchiasis OR Clonorchiosis OR Clonorchiasis OR Paragonimosis OR Paragonimiasis OR Metagonimus OR Heterophyiasis OR Fluke OR Flukes OR Trematode OR Trematodes OR Fasciola OR Fasciolopsis OR Opisthorchis OR Clonorchis OR Paragonimus OR "Minute intestinal fluke" OR "Minute intestinal flukes" OR "Haplorchis pumilio" OR "Metagonimus yokogawai" OR "Heterophyes" OR Diphyllobothriosis OR Diphyllobothriasis OR Bothriocephalosis OR Bothriocephaliasis OR Diphyllobothrium OR Bothriocephalus OR "Broad tapeworm" OR "Broad tapeworms" OR "Fish tapeworm" OR "Fish tapeworms" OR Cysticercosis OR Neurocysticercosis OR "Taenia solium" OR "Cystic echinococcosis" OR "Hydatid disease" OR	42 (10/9)	2 (1/23) *Dates limited to 10/9/22-1/31/24; Same filters

"Hydatid diseases" OR Hydatidosis OR "Echinococcus granulosus" OR "Hydatid cyst" OR "Hydatid cysts" OR "Alveolar echinococcosis" OR "Alveolar hydatidosis" OR "Echinococcus multilocularis") AND (Florid* OR "Southeastern United States”))<br><br>**FILTERS:** Scholarly journals only		
**Environmental Studies and Policy**	**2022 Results**	**2024 Results**
Keyword: "mammal" AND<br>Keyword: "parasite" OR<br>Keyword: “zoonotic diseases” OR<br>Keyword: “zoonotic parasites” AND<br>Keyword: florid* OR<br>Keyword: "southeastern united states"<br><br>**FILTERS:** Academic journals only; Peer-Reviewed journals	1,826 (10/10)	151 (1/23) *Dates limited to 10/11/22-1/31/24; Same filters
**Google Scholar**	**2022 Results**	**2024 Results**
allintitle: (parasite OR "zoonotic disease" OR "zoonotic diseases" OR "zoonotic parasites" OR "zoonotic enteric parasite") AND (Florida OR Floridian OR "Southeastern United States")<br><br>**FILTERS:** (none)	43 (10/9)	4 (1/23) *Dates limited to 2022-2024; Same filters
**Health and Medicine**	**2022 Results**	**2024 Results**
ab((armadillo* OR “nine banded armadillo” OR “nine-banded armadillo” OR “long nosed armadillo” OR “long-nosed armadillo” OR “armadillo nueve bandas” OR “dasypus novemcinctus” OR “dasypus novemcinctus aequatorialis” OR “dasypus novemcinctus fenestratus” OR “dasypus novemcinctus hoplites” OR “dasypus novemcinctus mexianae” OR “dasypus novemcinctus mexicanus” OR “dasypus novemcinctus novemcinctus” OR Opossum* OR possum* OR “virginia possum” OR “virginia possums” OR “virginia opossum” OR “virginia opossums” OR “didelphis virginiana” OR raccoon* OR “common raccoon” OR “common racoons” OR “northern raccoon” OR “procyon lotor” OR “procyon lotor auspicatus” OR “procyon lotor elucus” OR “procyon lotor excelsus” OR “procyon lotor fuscipes” OR “procyon lotor gloveralleni” OR “procyon lotor grinnelli” OR “procyon lotor hernandezii” OR “procyon lotor hirtus” OR “procyon lotor incautus” OR “procyon lotor inesperatus” OR “procyon lotor insularis” OR “procyon lotor litoreus” OR “procyon lotor lotor” OR “procyon lotor marinus” OR “procyon lotor maynardi” OR “procyon lotor megalodous” OR “procyon lotor pacificus” OR “procyon lotor pallidus” OR “procyon lotor psora” OR “procyon lotor pumilus” OR “procyon lotor simus” OR “procyon lotor vancouverensis” OR mole OR moles OR “eastern mole” OR “eastern moles” OR “scalopus aquaticus” OR “englewood mole” OR “scalopus aquaticus bassi” OR “scalopus aquaticus anastasae” OR “scalopus aquaticus aquaticus” OR bat OR bats OR “brazilian free-tailed bat” OR “mexican free-tailed bat” OR “tadarida brasiliensis” OR “tadarida brasiliensis Mexicana” OR “tadarida brasiliensis cynocephala” OR “eastern cotton tail” OR “eastern cottontail” OR “florida cottontail” OR “florida cotton tail” OR “sylvilagus floridanus” OR cottontail* OR “gray squirrel” OR “gray squirrels” OR “eastern gray squirrel” OR “eastern gray squirrels” OR “sciurus carolinensis” OR “sciurus carolinensis carolinensis” OR “sciurus carolinensis extimus” OR “sciurus carolinensis fuliginosus” OR “sciurus carolinensis hypophaeus” OR “red fox” OR “red foxes” OR “canis vulpes” OR vulpes OR “gray fox” OR “gray foxes” OR “common gray fox” OR “urocyon cinereoargenteus” OR coyote* OR “canis latrans” OR bobcat* OR “lynx rufus floridanus” OR “wildcat” OR “bay lynx” OR “lynx cat” OR “pallid lynx”) AND ("Zoonotic enteric pathogen" OR "Zoonotic enteric pathogens" OR "Zoonotic enteric parasite" OR "Zoonotic enteric parasites" OR "Zoonotic parasite" OR "Zoonotic parasites" OR "Animal-to-Human parasite" OR "Animal-to-Human parasites" OR "Foodborne parasite" OR "Foodborne parasites" OR "Waterborne parasite" OR "Waterborne parasites" OR "Water-related	47 (10/9)	3 (1/23) *Dates limited to 10/9/22-1/31/24; Same filters

parasite" OR "Water-related parasites" OR "Enteric parasite" OR "Enteric parasites" OR parasitism OR "Zoonotic intestinal helminth infection" OR "Zoonotic intestinal helminth infections" OR Helminthosis OR Helminthiasis OR Ascarosis OR Ascariasis OR Ancylostomosis OR Ancylostomiasis OR Trichuriosis OR Trichuriasis OR Strongyloidosis OR Strongyloidiasis OR Helminth* OR Ascaris OR Ancylostoma OR Hookworm* OR Trichuris OR Strongyloides OR Alaria OR "rat lungworm" OR "rat lungworms" OR "Echinostoma" OR "Lagochilascaris minor" OR "Zoonotic trypanosomosis" OR Trypanosomiasis OR Chagas OR "Trypanosoma cruzi" OR "Zoonotic intestinal protozoal infection" OR "Zoonotic intestinal protozoal infections" OR Protozoosis OR Protozoasis OR Giardiosis OR Giardiasis OR Cryptosporidiosis OR Blastocystosis OR Sarcocystosis OR Cyclosporiasis OR Cyclospora OR Amoebiasis OR "Amoebic dysentery" OR Entamoeba OR Balantidosis OR Protozoa OR Giardia OR Cryptosporidium OR Blastocystis OR Sarcocystis OR "Cyclospora cayetanensis" OR "Entamoeba histolytica" OR "Balantidium coli" OR Trichinellosis OR Trichinosis OR Trichinella OR Toxoplasmosis OR TORCH OR Toxoplasma OR Toxocarosis OR Toxocariasis OR Toxocariosis OR "Larva migrans" OR Toxocara OR Taeniosis OR Taeniasis OR Tapeworm OR Tapeworms OR Taenia OR "Foodborne trematodosis" OR trematodosis OR Trematodiasis OR Fasciolosis OR Fascioliosis OR Fasciolasis OR Fascioliasis OR Distomatosis OR Fasciolopsiasis OR Fasciolopsiosis OR Opisthorchosis OR Opisthorchiasis OR Clonorchiosis OR Clonorchiasis OR Paragonimosis OR Paragonimiasis OR Metagonimus OR Heterophyiasis OR Fluke OR Flukes OR Trematode OR Trematodes OR Fasciola OR Fasciolopsis OR Opisthorchis OR Clonorchis OR Paragonimus OR "Minute intestinal fluke" OR "Minute intestinal flukes" OR "Haplorchis pumilio" OR "Metagonimus yokogawai" OR "Heterophyes" OR Diphyllobothriosis OR Diphyllobothriasis OR Bothriocephalosis OR Bothriocephaliasis OR Diphyllobothrium OR Bothriocephalus OR "Broad tapeworm" OR "Broad tapeworms" OR "Fish tapeworm" OR "Fish tapeworms" OR Cysticercosis OR Neurocysticercosis OR "Taenia solium" OR "Cystic echinococcosis" OR "Hydatid disease" OR "Hydatid diseases" OR Hydatidosis OR "Echinococcus granulosus" OR "Hydatid cyst" OR "Hydatid cysts" OR "Alveolar echinococcosis" OR "Alveolar hydatidosis" OR "Echinococcus multilocularis") AND (Florid* OR "Southeastern United States"))<br><br>**FILTERS:** Scholarly Journals		
**MEDLINE**	**2022 Results**	**2024 Results**
ab((armadillo* OR "nine banded armadillo" OR "nine-banded armadillo" OR "long nosed armadillo" OR "long-nosed armadillo" OR "armadillo nueve bandas" OR "dasypus novemcinctus" OR "dasypus novemcinctus aequatorialis" OR "dasypus novemcinctus fenestratus" OR "dasypus novemcinctus hoplites" OR "dasypus novemcinctus mexianae" OR "dasypus novemcinctus mexicanus" OR "dasypus novemcinctus novemcinctus" OR Opossum* OR possum* OR "virginia possum" OR "virginia possums" OR "virginia opossum" OR "virginia opossums" OR "didelphis virginiana" OR raccoon* OR "common raccoon" OR "common racoons" OR "northern raccoon" OR "procyon lotor" OR "procyon lotor auspicatus" OR "procyon lotor elucus" OR "procyon lotor excelsus" OR "procyon lotor fuscipes" OR "procyon lotor gloveralleni" OR "procyon lotor grinnelli" OR "procyon lotor hernandezii" OR "procyon lotor hirtus" OR "procyon lotor incautus" OR "procyon lotor inesperatus" OR "procyon lotor insularis" OR "procyon lotor litoreus" OR "procyon lotor lotor" OR "procyon lotor marinus" OR "procyon lotor maynardi" OR "procyon lotor megalodous" OR "procyon lotor pacificus" OR "procyon lotor pallidus" OR "procyon lotor psora" OR "procyon lotor pumilus" OR "procyon lotor simus" OR "procyon lotor vancouverensis" OR mole OR moles OR "eastern mole" OR "eastern moles" OR "scalopus aquaticus" OR "englewood mole" OR "scalopus aquaticus bassi" OR "scalopus aquaticus anastasae" OR "scalopus aquaticus aquaticus" OR bat OR bats OR "brazilian free-tailed bat" OR "mexican free-tailed bat" OR "tadarida brasiliensis" OR "tadarida brasiliensis Mexicana" OR "tadarida brasiliensis cynocephala" OR "eastern cotton tail" OR "eastern cottontail" OR "florida cottontail" OR "florida cotton tail" OR "sylvilagus floridanus" OR cottontail* OR "gray squirrel" OR "gray squirrels" OR "eastern gray squirrel" OR "eastern gray squirrels" OR "sciurus carolinensis" OR "sciurus carolinensis carolinensis" OR "sciurus carolinensis extimus" OR "sciurus carolinensis fuliginosus" OR "sciurus carolinensis hypophaeus" OR "red fox" OR "red foxes" OR "canis vulpes" OR vulpes OR "gray fox" OR "gray foxes" OR "common gray fox" OR "urocyon cinereoargenteus" OR coyote* OR "canis latrans" OR bobcat* OR "lynx rufus floridanus" OR "wildcat" OR "bay lynx" OR "lynx cat" OR "pallid lynx") AND ("Zoonotic enteric pathogen" OR "Zoonotic enteric pathogens" OR "Zoonotic enteric parasite" OR "Zoonotic enteric parasites" OR "Zoonotic parasite" OR "Zoonotic parasites" OR "Animal-to-Human parasite" OR "Animal-to-Human parasites" OR "Foodborne parasite" OR "Foodborne parasites" OR "Waterborne parasite" OR "Waterborne parasites" OR "Water-related parasite" OR "Water-related parasites" OR "Enteric parasite" OR "Enteric parasites" OR parasitism OR "Zoonotic intestinal helminth infection" OR "Zoonotic intestinal helminth infections" OR Helminthosis OR Helminthiasis OR Ascarosis OR Ascariasis OR Ancylostomosis OR Ancylostomiasis OR Trichuriosis OR Trichuriasis OR Strongyloidosis OR Strongyloidiasis OR Helminth* OR Ascaris OR Ancylostoma OR Hookworm* OR Trichuris OR Strongyloides OR Alaria OR "rat lungworm" OR "rat lungworms" OR "Echinostoma" OR "Lagochilascaris minor" OR "Zoonotic trypanosomosis" OR Trypanosomiasis OR Chagas OR "Trypanosoma cruzi" OR "Zoonotic intestinal protozoal infection" OR "Zoonotic intestinal protozoal infections"	47 (10/9)	3 (1/23) *Dates limited to 10/9/22-1/31/24

OR Protozoosis OR Protozoasis OR Giardiosis OR Giardiasis OR Cryptosporidiosis OR Blastocystosis OR Sarcocystosis OR Cyclosporiasis OR Cyclospora OR Amoebiasis OR "Amoebic dysentery" OR Entamoeba OR Balantidosis OR Protozoa OR Giardia OR Cryptosporidium OR Blastocystis OR Sarcocystis OR "Cyclospora cayetanensis" OR "Entamoeba histolytica" OR "Balantidium coli" OR Trichinellosis OR Trichinosis OR Trichinella OR Toxoplasmosis OR TORCH OR Toxoplasma OR Toxocarosis OR Toxocariasis OR Toxocariosis OR "Larva migrans" OR Toxocara OR Taeniosis OR Taeniasis OR Tapeworm OR Tapeworms OR Taenia OR "Foodborne trematodosis" OR trematodosis OR Trematodiasis OR Fasciolosis OR Fascioliosis OR Fasciolasis OR Fascioliasis OR Distomatosis OR Fasciolopsiasis OR Fasciolopsiosis OR Opisthorchosis OR Opisthorchiasis OR Clonorchiosis OR Clonorchiasis OR Paragonimosis OR Paragonimiasis OR Metagonimus OR Heterophyiasis OR Fluke OR Flukes OR Trematode OR Trematodes OR Fasciola OR Fasciolopsis OR Opisthorchis OR Clonorchis OR Paragonimus OR "Minute intestinal fluke" OR "Minute intestinal flukes" OR "Haplorchis pumilio" OR "Metagonimus yokogawai" OR "Heterophyes" OR Diphyllobothriosis OR Diphyllobothriasis OR Bothriocephalosis OR Bothriocephaliasis OR Diphyllobothrium OR Bothriocephalus OR "Broad tapeworm" OR "Broad tapeworms" OR "Fish tapeworm" OR "Fish tapeworms" OR Cysticercosis OR Neurocysticercosis OR "Taenia solium" OR "Cystic echinococcosis" OR "Hydatid disease" OR "Hydatid diseases" OR Hydatidosis OR "Echinococcus granulosus" OR "Hydatid cyst" OR "Hydatid cysts" OR "Alveolar echinococcosis" OR "Alveolar hydatidosis" OR "Echinococcus multilocularis") AND (Florid* OR "Southeastern United States”))<br><br>**FILTERS:** (none)		
**Nursing and Allied Health Outcomes**	**2022 Results**	**2024 Results**
Keyword: "mammal" AND<br>Keyword: "parasite" OR<br>Keyword: “zoonotic diseases” OR<br>Keyword: “zoonotic parasites” AND<br>Keyword: florid* OR<br>Keyword: "southeastern united states"<br><br>**FILTERS:** Academic journals only; Peer-Reviewed journals	1,544 (10/10)	185 (1/23) *Dates limited to 10/10/22-1/31/24; Same filters
**SAGE Journals**	**2022 Results**	**2024 Results**
((armadillo* OR “nine banded armadillo” OR “nine-banded armadillo” OR “long nosed armadillo” OR “long-nosed armadillo” OR “armadillo nueve bandas” OR “dasypus novemcinctus” OR “dasypus novemcinctus aequatorialis” OR “dasypus novemcinctus fenestratus” OR “dasypus novemcinctus hoplites” OR “dasypus novemcinctus mexianae” OR “dasypus novemcinctus mexicanus” OR “dasypus novemcinctus novemcinctus” OR Opossum* OR possum* OR “virginia possum” OR “virginia possums” OR “virginia opossum” OR “virginia opossums” OR “didelphis virginiana” OR raccoon* OR “common raccoon” OR “common racoons” OR “northern raccoon” OR “procyon lotor” OR “procyon lotor auspicatus” OR “procyon lotor elucus” OR “procyon lotor excelsus” OR “procyon lotor fuscipes” OR “procyon lotor gloveralleni” OR “procyon lotor grinnelli” OR “procyon lotor hernandezii” OR “procyon lotor hirtus” OR “procyon lotor incautus” OR “procyon lotor inesperatus” OR “procyon lotor insularis” OR “procyon lotor litoreus” OR “procyon lotor lotor” OR “procyon lotor marinus” OR “procyon lotor maynardi” OR “procyon lotor megalodous” OR “procyon lotor pacificus” OR “procyon lotor pallidus” OR “procyon lotor psora” OR “procyon lotor pumilus” OR “procyon lotor simus” OR “procyon lotor vancouverensis” OR mole OR moles OR “eastern mole” OR “eastern moles” OR “scalopus aquaticus” OR “englewood mole” OR “scalopus aquaticus bassi” OR “scalopus aquaticus anastasae” OR “scalopus aquaticus aquaticus” OR bat OR bats OR “brazilian free-tailed bat” OR “mexican free-tailed bat” OR “tadarida brasiliensis” OR “tadarida brasiliensis Mexicana” OR “tadarida brasiliensis cynocephala” OR “eastern cotton tail” OR “eastern cottontail” OR “florida cottontail” OR “florida cotton tail” OR “sylvilagus floridanus” OR cottontail* OR “gray squirrel” OR “gray squirrels” OR “eastern gray squirrel” OR “eastern gray squirrels” OR “sciurus carolinensis” OR “sciurus carolinensis carolinensis” OR “sciurus carolinensis extimus” OR “sciurus carolinensis fuliginosus” OR “sciurus carolinensis hypophaeus” OR “red fox” OR “red foxes” OR “canis vulpes” OR vulpes OR “gray fox” OR “gray foxes” OR “common gray fox” OR “urocyon cinereoargenteus” OR coyote* OR “canis latrans” OR bobcat* OR “lynx rufus floridanus” OR “wildcat” OR “bay lynx” OR “lynx cat” OR “pallid lynx”) AND ("Zoonotic enteric pathogen" OR "Zoonotic enteric pathogens" OR "Zoonotic enteric parasite" OR "Zoonotic enteric parasites" OR "Zoonotic parasite" OR "Zoonotic parasites" OR "Animal-to-Human parasite" OR "Animal-to-Human parasites" OR "Foodborne parasite" OR "Foodborne parasites" OR	2 (10/9)	3 (1/23) *Dates limited to 10/2022-1/2024; Filter: Research Article

"Waterborne parasite" OR "Waterborne parasites" OR "Water-related parasite" OR "Water-related parasites" OR "Enteric parasite" OR "Enteric parasites" OR parasitism OR "Zoonotic intestinal helminth infection" OR "Zoonotic intestinal helminth infections" OR Helminthosis OR Helminthiasis OR Ascarosis OR Ascariasis OR Ancylostomosis OR Ancylostomiasis OR Trichuriosis OR Trichuriasis OR Strongyloidosis OR Strongyloidiasis OR Helminth* OR Ascaris OR Ancylostoma OR Hookworm* OR Trichuris OR Strongyloides OR Alaria OR "rat lungworm" OR "rat lungworms" OR "Echinostoma" OR "Lagochilascaris minor" OR "Zoonotic trypanosomosis" OR Trypanosomiasis OR Chagas OR "Trypanosoma cruzi" OR "Zoonotic intestinal protozoal infection" OR "Zoonotic intestinal protozoal infections" OR Protozoosis OR Protozoasis OR Giardiosis OR Giardiasis OR Cryptosporidiosis OR Blastocystosis OR Sarcocystosis OR Cyclosporiasis OR Cyclospora OR Amoebiasis OR "Amoebic dysentery" OR Entamoeba OR Balantidosis OR Protozoa OR Giardia OR Cryptosporidium OR Blastocystis OR Sarcocystis OR "Cyclospora cayetanensis" OR "Entamoeba histolytica" OR "Balantidium coli" OR Trichinellosis OR Trichinosis OR Trichinella OR Toxoplasmosis OR TORCH OR Toxoplasma OR Toxocarosis OR Toxocariasis OR Toxocariosis OR "Larva migrans" OR Toxocara OR Taeniosis OR Taeniasis OR Tapeworm OR Tapeworms OR Taenia OR "Foodborne trematodosis" OR trematodosis OR Trematodiasis OR Fasciolosis OR Fascioliosis OR Fasciolasis OR Fascioliasis OR Distomatosis OR Fasciolopsiasis OR Fasciolopsiosis OR Opisthorchosis OR Opisthorchiasis OR Clonorchiosis OR Clonorchiasis OR Paragonimosis OR Paragonimiasis OR Metagonimus OR Heterophyiasis OR Fluke OR Flukes OR Trematode OR Trematodes OR Fasciola OR Fasciolopsis OR Opisthorchis OR Clonorchis OR Paragonimus OR "Minute intestinal fluke" OR "Minute intestinal flukes" OR "Haplorchis pumilio" OR "Metagonimus yokogawai" OR "Heterophyes" OR Diphyllobothriosis OR Diphyllobothriasis OR Bothriocephalosis OR Bothriocephaliasis OR Diphyllobothrium OR Bothriocephalus OR "Broad tapeworm" OR "Broad tapeworms" OR "Fish tapeworm" OR "Fish tapeworms" OR Cysticercosis OR Neurocysticercosis OR "Taenia solium" OR "Cystic echinococcosis" OR "Hydatid disease" OR "Hydatid diseases" OR Hydatidosis OR "Echinococcus granulosus" OR "Hydatid cyst" OR "Hydatid cysts" OR "Alveolar echinococcosis" OR "Alveolar hydatidosis" OR "Echinococcus multilocularis") AND (Florid* OR "Southeastern United States”))<br><br>**FILTERS:** (none)		
**Science Direct**	**2022 Results**	**2024 Results**
<u>Find articles with these terms:</u> (Florida OR Floridian OR "Southeastern United States")<br><u>Title, abstract, and keywords:</u> (parasite OR "zoonotic disease" OR "zoonotic diseases" OR "zoonotic parasites" OR "zoonotic enteric parasite")<br><br>**FILTERS:** Article Types: Research Articles, Case Reports, Data Articles, Short Communication, Other	1,619 (10/9)	132 (1/24) *Dates limited to 10/2022-1/2024; Same filters
**TOXLINE**	**2022 Results**	**2024 Results**
AB((armadillo* OR “nine banded armadillo” OR “nine-banded armadillo” OR “long nosed armadillo” OR “long-nosed armadillo” OR “armadillo nueve bandas” OR “dasypus novemcinctus” OR “dasypus novemcinctus aequatorialis” OR “dasypus novemcinctus fenestratus” OR “dasypus novemcinctus hoplites” OR “dasypus novemcinctus mexianae” OR “dasypus novemcinctus mexicanus” OR “dasypus novemcinctus novemcinctus” OR Opossum* OR possum* OR “virginia possum” OR “virginia possums” OR “virginia opossum” OR “virginia opossums” OR “didelphis virginiana” OR raccoon* OR “common raccoon” OR “common racoons” OR “northern raccoon” OR “procyon lotor” OR “procyon lotor auspicatus” OR “procyon lotor elucus” OR “procyon lotor excelsus” OR “procyon lotor fuscipes” OR “procyon lotor gloveralleni” OR “procyon lotor grinnelli” OR “procyon lotor hernandezii” OR “procyon lotor hirtus” OR “procyon lotor incautus” OR “procyon lotor inesperatus” OR “procyon lotor insularis” OR “procyon lotor litoreus” OR “procyon lotor lotor” OR “procyon lotor marinus” OR “procyon lotor maynardi” OR “procyon lotor megalodous” OR “procyon lotor pacificus” OR “procyon lotor pallidus” OR “procyon lotor psora” OR “procyon lotor pumilus” OR “procyon lotor simus” OR “procyon lotor vancouverensis” OR mole OR moles OR “eastern mole” OR “eastern moles” OR “scalopus aquaticus” OR “englewood mole” OR “scalopus aquaticus bassi” OR “scalopus aquaticus anastasae” OR “scalopus aquaticus aquaticus” OR bat OR bats OR “brazilian free-tailed bat” OR “mexican free-tailed bat” OR “tadarida brasiliensis” OR “tadarida brasiliensis Mexicana” OR “tadarida brasiliensis cynocephala” OR “eastern cotton tail” OR “eastern cottontail” OR “florida cottontail” OR “florida cotton tail” OR “sylvilagus floridanus” OR cottontail* OR “gray squirrel” OR “gray squirrels” OR “eastern gray squirrel” OR “eastern gray squirrels” OR “sciurus carolinensis” OR “sciurus carolinensis carolinensis” OR “sciurus carolinensis extimus”	4 (10/9)	0 (1/24) *Dates limited to 10/9/2022 - 1/31/2024 ; Same filters

OR “sciurus carolinensis fuliginosus” OR “sciurus carolinensis hypophaeus” OR “red fox” OR “red foxes” OR “canis vulpes” OR vulpes OR “gray fox” OR “gray foxes” OR “common gray fox” OR “urocyon cinereoargenteus” OR coyote* OR “canis latrans” OR bobcat* OR “lynx rufus floridanus” OR “wildcat” OR “bay lynx” OR “lynx cat” OR “pallid lynx”) AND ("Zoonotic enteric pathogen" OR "Zoonotic enteric pathogens" OR "Zoonotic enteric parasite" OR "Zoonotic enteric parasites" OR "Zoonotic parasite" OR "Zoonotic parasites" OR "Animal-to-Human parasite" OR "Animal-to-Human parasites" OR "Foodborne parasite" OR "Foodborne parasites" OR "Waterborne parasite" OR "Waterborne parasites" OR "Water-related parasite" OR "Water-related parasites" OR "Enteric parasite" OR "Enteric parasites" OR parasitism OR "Zoonotic intestinal helminth infection" OR "Zoonotic intestinal helminth infections" OR Helminthosis OR Helminthiasis OR Ascarosis OR Ascariasis OR Ancylostomosis OR Ancylostomiasis OR Trichuriosis OR Trichuriasis OR Strongyloidosis OR Strongyloidiasis OR Helminth* OR Ascaris OR Ancylostoma OR Hookworm* OR Trichuris OR Strongyloides OR Alaria OR "rat lungworm" OR "rat lungworms" OR "Echinostoma" OR "Lagochilascaris minor" OR "Zoonotic trypanosomosis" OR Trypanosomiasis OR Chagas OR "Trypanosoma cruzi" OR "Zoonotic intestinal protozoal infection" OR "Zoonotic intestinal protozoal infections" OR Protozoosis OR Protozoasis OR Giardiosis OR Giardiasis OR Cryptosporidiosis OR Blastocystosis OR Sarcocystosis OR Cyclosporiasis OR Cyclospora OR Amoebiasis OR "Amoebic dysentery" OR Entamoeba OR Balantidosis OR Protozoa OR Giardia OR Cryptosporidium OR Blastocystis OR Sarcocystis OR "Cyclospora cayetanensis" OR "Entamoeba histolytica" OR "Balantidium coli" OR Trichinellosis OR Trichinosis OR Trichinella OR Toxoplasmosis OR TORCH OR Toxoplasma OR Toxocarosis OR Toxocariasis OR Toxocariosis OR "Larva migrans" OR Toxocara OR Taeniosis OR Taeniasis OR Tapeworm OR Tapeworms OR Taenia OR "Foodborne trematodosis" OR trematodosis OR Trematodiasis OR Fasciolosis OR Fascioliosis OR Fasciolasis OR Fascioliasis OR Distomatosis OR Fasciolopsiasis OR Fasciolopsiosis OR Opisthorchosis OR Opisthorchiasis OR Clonorchiosis OR Clonorchiasis OR Paragonimosis OR Paragonimiasis OR Metagonimus OR Heterophyiasis OR Fluke OR Flukes OR Trematode OR Trematodes OR Fasciola OR Fasciolopsis OR Opisthorchis OR Clonorchis OR Paragonimus OR "Minute intestinal fluke" OR "Minute intestinal flukes" OR "Haplorchis pumilio" OR "Metagonimus yokogawai" OR "Heterophyes" OR Diphyllobothriosis OR Diphyllobothriasis OR Bothriocephalosis OR Bothriocephaliasis OR Diphyllobothrium OR Bothriocephalus OR "Broad tapeworm" OR "Broad tapeworms" OR "Fish tapeworm" OR "Fish tapeworms" OR Cysticercosis OR Neurocysticercosis OR "Taenia solium" OR "Cystic echinococcosis" OR "Hydatid disease" OR "Hydatid diseases" OR Hydatidosis OR "Echinococcus granulosus" OR "Hydatid cyst" OR "Hydatid cysts" OR "Alveolar echinococcosis" OR "Alveolar hydatidosis" OR "Echinococcus multilocularis") AND (Florid* OR "Southeastern United States”))<br><br>**FILTERS:** (none)		
**Web of Science Core Collection**	**2022 Results**	**2024 Results**
Topic= ((armadillo* OR “nine banded armadillo” OR “nine-banded armadillo” OR “long nosed armadillo” OR “long-nosed armadillo” OR “armadillo nueve bandas” OR “dasypus novemcinctus” OR “dasypus novemcinctus aequatorialis” OR “dasypus novemcinctus fenestratus” OR “dasypus novemcinctus hoplites” OR “dasypus novemcinctus mexianae” OR “dasypus novemcinctus mexicanus” OR “dasypus novemcinctus novemcinctus” OR Opossum* OR possum* OR “virginia possum” OR “virginia possums” OR “virginia opossum” OR “virginia opossums” OR “didelphis virginiana” OR raccoon* OR “common raccoon” OR “common racoons” OR “northern raccoon” OR “procyon lotor” OR “procyon lotor auspicatus” OR “procyon lotor elucus” OR “procyon lotor excelsus” OR “procyon lotor fuscipes” OR “procyon lotor gloveralleni” OR “procyon lotor grinnelli” OR “procyon lotor hernandezii” OR “procyon lotor hirtus” OR “procyon lotor incautus” OR “procyon lotor inesperatus” OR “procyon lotor insularis” OR “procyon lotor litoreus” OR “procyon lotor lotor” OR “procyon lotor marinus” OR “procyon lotor maynardi” OR “procyon lotor megalodous” OR “procyon lotor pacificus” OR “procyon lotor pallidus” OR “procyon lotor psora” OR “procyon lotor pumilus” OR “procyon lotor simus” OR “procyon lotor vancouverensis” OR mole OR moles OR “eastern mole” OR “eastern moles” OR “scalopus aquaticus” OR “englewood mole” OR “scalopus aquaticus bassi” OR “scalopus aquaticus anastasae” OR “scalopus aquaticus aquaticus” OR bat OR bats OR “brazilian free-tailed bat” OR “mexican free-tailed bat” OR “tadarida brasiliensis” OR “tadarida brasiliensis Mexicana” OR “tadarida brasiliensis cynocephala” OR “eastern cotton tail” OR “eastern cottontail” OR “florida cottontail” OR “florida cotton tail” OR “sylvilagus floridanus” OR cottontail* OR “gray squirrel” OR “gray squirrels” OR “eastern gray squirrel” OR “eastern gray squirrels” OR “sciurus carolinensis” OR “sciurus carolinensis carolinensis” OR “sciurus carolinensis extimus” OR “sciurus carolinensis fuliginosus” OR “sciurus carolinensis hypophaeus” OR “red fox” OR “red foxes” OR “canis vulpes” OR vulpes OR “gray fox” OR “gray foxes” OR “common gray fox” OR “urocyon cinereoargenteus” OR coyote* OR “canis latrans” OR bobcat* OR “lynx rufus floridanus” OR “wildcat” OR “bay lynx” OR “lynx cat” OR “pallid lynx”) AND ("Zoonotic enteric pathogen" OR "Zoonotic enteric pathogens" OR "Zoonotic enteric parasite" OR "Zoonotic enteric parasites" OR "Zoonotic parasite" OR "Zoonotic parasites" OR "Animal-to-Human parasite" OR "Animal-to-Human parasites" OR "Foodborne parasite" OR "Foodborne parasites" OR "Waterborne parasite" OR "Waterborne parasites" OR "Water-related	97 (10/6	5 (1/24)<br>*Dates limited to 10/6/2022<br>-<br>1/31/2024

parasite" OR "Water-related parasites" OR "Enteric parasite" OR "Enteric parasites" OR parasitism OR "Zoonotic intestinal helminth infection" OR "Zoonotic intestinal helminth infections" OR Helminthosis OR Helminthiasis OR Ascarosis OR Ascariasis OR Ancylostomosis OR Ancylostomiasis OR Trichuriosis OR Trichuriasis OR Strongyloidosis OR Strongyloidiasis OR Helminth* OR Ascaris OR Ancylostoma OR Hookworm* OR Trichuris OR Strongyloides OR Alaria OR "rat lungworm" OR "rat lungworms" OR "Echinostoma" OR "Lagochilascaris minor" OR "Zoonotic trypanosomosis" OR Trypanosomiasis OR Chagas OR "Trypanosoma cruzi" OR "Zoonotic intestinal protozoal infection" OR "Zoonotic intestinal protozoal infections" OR Protozoosis OR Protozoasis OR Giardiosis OR Giardiasis OR Cryptosporidiosis OR Blastocystosis OR Sarcocystosis OR Cyclosporiasis OR Cyclospora OR Amoebiasis OR "Amoebic dysentery" OR Entamoeba OR Balantidosis OR Protozoa OR Giardia OR Cryptosporidium OR Blastocystis OR Sarcocystis OR "Cyclospora cayetanensis" OR "Entamoeba histolytica" OR "Balantidium coli" OR Trichinellosis OR Trichinosis OR Trichinella OR Toxoplasmosis OR TORCH OR Toxoplasma OR Toxocarosis OR Toxocariasis OR Toxocariosis OR "Larva migrans" OR Toxocara OR Taeniosis OR Taeniasis OR Tapeworm OR Tapeworms OR Taenia OR "Foodborne trematodosis" OR trematodosis OR Trematodiasis OR Fasciolosis OR Fascioliosis OR Fasciolasis OR Fascioliasis OR Distomatosis OR Fasciolopsiasis OR Fasciolopsiosis OR Opisthorchosis OR Opisthorchiasis OR Clonorchiosis OR Clonorchiasis OR Paragonimosis OR Paragonimiasis OR Metagonimus OR Heterophyiasis OR Fluke OR Flukes OR Trematode OR Trematodes OR Fasciola OR Fasciolopsis OR Opisthorchis OR Clonorchis OR Paragonimus OR "Minute intestinal fluke" OR "Minute intestinal flukes" OR "Haplorchis pumilio" OR "Metagonimus yokogawai" OR "Heterophyes" OR Diphyllobothriosis OR Diphyllobothriasis OR Bothriocephalosis OR Bothriocephaliasis OR Diphyllobothrium OR Bothriocephalus OR "Broad tapeworm" OR "Broad tapeworms" OR "Fish tapeworm" OR "Fish tapeworms" OR Cysticercosis OR Neurocysticercosis OR "Taenia solium" OR "Cystic echinococcosis" OR "Hydatid disease" OR "Hydatid diseases" OR Hydatidosis OR "Echinococcus granulosus" OR "Hydatid cyst" OR "Hydatid cysts" OR "Alveolar echinococcosis" OR "Alveolar hydatidosis" OR "Echinococcus multilocularis") AND (Florid* OR "Southeastern United States")) <br>**FILTERS:** (none)		
**Total**	9696	867
